# CCAAT/Enhancer-Binding Proteins in Fibrosis: Complex Roles Beyond Conventional Understanding

**DOI:** 10.34133/2022/9891689

**Published:** 2022-10-03

**Authors:** Lexun Wang, Jiaojiao Feng, Yanyue Deng, Qianqian Yang, Quxing Wei, Dewei Ye, Xianglu Rong, Jiao Guo

**Affiliations:** ^1^Guangdong Metabolic Diseases Research Center of Integrated Chinese and Western Medicine, China; ^2^Key Laboratory of Glucolipid Metabolic Disorder, Ministry of Education of China, China; ^3^Guangdong Key Laboratory of Metabolic Disease Prevention and Treatment of Traditional Chinese Medicine, China; ^4^Institute of Chinese Medicine, Guangdong Pharmaceutical University, Guangzhou, China

## Abstract

CCAAT/enhancer-binding proteins (C/EBPs) are a family of at least six identified transcription factors that contain a highly conserved basic leucine zipper domain and interact selectively with duplex DNA to regulate target gene expression. C/EBPs play important roles in various physiological processes, and their abnormal function can lead to various diseases. Recently, accumulating evidence has demonstrated that aberrant C/EBP expression or activity is closely associated with the onset and progression of fibrosis in several organs and tissues. During fibrosis, various C/EBPs can exert distinct functions in the same organ, while the same C/EBP can exert distinct functions in different organs. Modulating C/EBP expression or activity could regulate various molecular processes to alleviate fibrosis in multiple organs; therefore, novel C/EBPs-based therapeutic methods for treating fibrosis have attracted considerable attention. In this review, we will explore the features of C/EBPs and their critical functions in fibrosis in order to highlight new avenues for the development of novel therapies targeting C/EBPs.

## 1. Introduction

Fibrosis, characterized by the excessive deposition of extracellular matrix (ECM) in the tissues, is not a disease but rather an outcome of the tissue repair response [1]. Fibrosis is a pathological hallmark of diseases in virtually any solid organ or tissue, which can be caused by diseases, physical and chemical stimulations, and trauma [2]. Multiple common diseases can lead to fibrosis, including diabetes, hypertension, myocardial infarction, heart failure, nonalcoholic steatohepatitis, hepatitis, idiopathic pulmonary disease, chronic kidney disease, scleroderma, and cancer. Persistent fibrosis can result in organ dysfunction and death. The annual incidence of fibrosis-related diseases is approximately 5% worldwide [3]. Moreover, fibrosis causes up to 45% of all deaths in the developed countries [1].

The inflammatory response plays a critical role in the initiation of fibrosis [2, 4–7]. In addition, the activation of ECM-producing cells is arguably a central event in fibrogenesis [8]. Although many cells can produce ECM, including 0fibroblasts, vascular smooth muscle cells, epithelial cells, and a subset of macrophages, activated fibroblasts (also referred to as myofibroblasts) are regarded as the principal ECM-producing cells as they generate numerous ECM components, including type I and III collagen [8–13]. Multiple complex molecular mechanisms are involved in fibrosis. For instance, transforming growth factor-*β* (TGF-*β*), connective tissue growth factor (CTGF), platelet-derived growth factor (PDGF), and integrins have been identified as important fibrosis regulators [14–17]. Although some cellular and molecular processes underlying fibrosis have been elucidated in the past decade, few effective therapeutic strategies and drugs have been developed that can specifically target fibrogenesis [2, 14, 18]. These facts highlight the need for a deeper understanding of the pathogenesis of fibrosis and conversion of this knowledge into novel prophylaxis and treatment strategies.

CCAAT/enhancer-binding proteins (C/EBPs) are a family of basic region leucine-zipper (bZIP) transcription factor that dimerizes through a highly conserved C-terminal ZIP domain and bind to DNA through an adjacent basic region. To date, six members of this family have been identified and named in chronological order of their discovery: C/EBP*α*, C/EBP*β*, C/EBP*γ*, C/EBP*δ*, C/EBP*ε*, and C/EBP*ζ* [19–22]. The N-terminal of C/EBP proteins is more varied than the C-terminal. C/EBP*α*, C/EBP*β*, C/EBP*δ*, and C/EBP*ε* carry both activation and regulatory domains in their N-terminals [19–22], whereas C/EBP*γ* and C/EBP*ζ* lack activation domains and instead repress gene transcription by building inactive heterodimers with other members or transcriptional factors [21, 23]. Two isoforms of C/EBP*α* and three isoforms of C/EBP*β* have been reported to function as activators or inhibitors depending on the number of activation or regulatory domains in N-terminal [20, 22]. In addition, C/EBP*α* expresses mainly in adipocytes, hepatocytes, and myeloid cells of hematopoietic system, whereas C/EBP*β* has been detected in the adipose tissue, heart, liver, and brain [22, 24]. C/EBP*γ* and C/EBP*ζ* ubiquitously express in most organs and tissues, but CEBP*δ* is expressed at low level in tissues and can be rapidly induced by stimuli [21, 25, 26]. C/EBP*ε* is exclusively detected in myeloid cells [19]. The differences in CEBP expression profiles determine their important and unique roles in different tissues and organs. C/EBPs have been reported to affect various physiological processes, such as hematopoiesis, adipogenesis, energy metabolism, innate and adaptive immunity, inflammation, cellular proliferation and differentiation, apoptosis, and autophagy [21, 23–29]. Consequently, aberrant C/EBP expression or activity can affect the occurrence and progression of various diseases, including cancers, Alzheimer's disease, pneumonia, and cardiac infraction [23, 27, 30–33]. Recently, an increasing number of studies have revealed that abnormal C/EBP expression and/or activation is closely related to the development of fibrosis in multiple organs [23–26, 28, 34–36]. For instance, protein levels of C/EBP*α* decrease in carbon tetrachloride- (CCl_4_-) induced fibrotic liver tissue, and overexpression of C/EBP*α* in the liver can alleviate CCl_4_-induced hepatic fibrosis [28, 37]. C/EBP*β* expression is inhibited in diabetic cardiomyopathy- (DCM-) induced fibrotic heart tissue, and overexpression of C/EBP*β* inhibits this cardiac fibrosis, while C/EBP*β* knockdown attenuates heart fibrotic pathology in rat models of experimental autoimmune myocarditis (EAM) [34, 38]. The overactivation of C/EBP*γ* induced by IL-1*β* inhibits IL-6 expression in lung epithelial cells, which indirectly suppresses lung fibrosis [25, 39]. In the animal models, the C/EBP*δ* protein levels increased in kidney tissues during renal fibrosis [26]. Liver fibrosis is significantly reduced in C/EBP*ζ*^−/−^ mice after a bile duct ligation operation, whereas C/EBP*ζ*^−/−^ mice develops greater fibrosis than wild type mice when given a high-fat diet [40, 41]. In general, during fibrotic progression of a given tissue, C/EBP*α* and C/EBP*γ* play a negative role in fibrogenesis, while C/EBP*β*, C/EBP*δ*, and C/EBP*ζ* have positive roles [25, 42–45]. A full overview of C/EBPs and their roles in fibrosis may be important to provide new therapeutic targets for treating fibrosis.

Here, we summarize the properties of C/EBP genes, proteins, and posttranslational modification (PTM). Then, we mainly review the crucial roles of C/EBPs in the fibrosis of different organs. Finally, we discuss current and future challenges in drug discovery and development of fibrosis therapies based on modulating C/EBP expression or activity.

## 2. Biological Features of C/EBPs

### 2.1. C/EBP Genes and mRNAs

**Figure 1:**
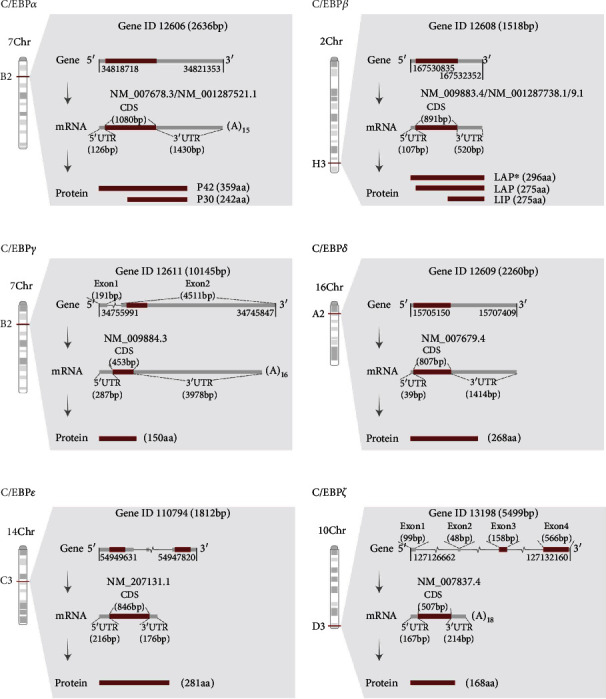
Schematic diagram of genes, mRNAs, and proteins of mouse C/EBP family members. The gene location, exons and introns, mRNA information, and protein isoforms of C/EBPs are shown (accessed September 9, 2022, National Center for Biotechnology Information).

Since the first C/EBP gene was identified and cloned from the rat liver tissue in 1988 [46], C/EBP genes have been cloned from various species and many of their proteins have been characterized and named independently, as summarized in Figure 1. It should be noted that C/EBP*ζ* (also called CBF, CBF2, HSP-CBF, and NOC1) coded by *Cebpz* (gene ID: 12607 for mouse and 10153 for human) is excluded from the CEBP family as it lacks the bZIP motif and has low homology with other C/EBP members and is known as C/EBP homologous protein (CHOP, also known as growth arrest and DNA damage-inducible protein 153 (GADD153)) [20, 21, 29, 31]. For consistency, we referred to CHOP as C/EBP*ζ* in this review.

**Figure 2:**
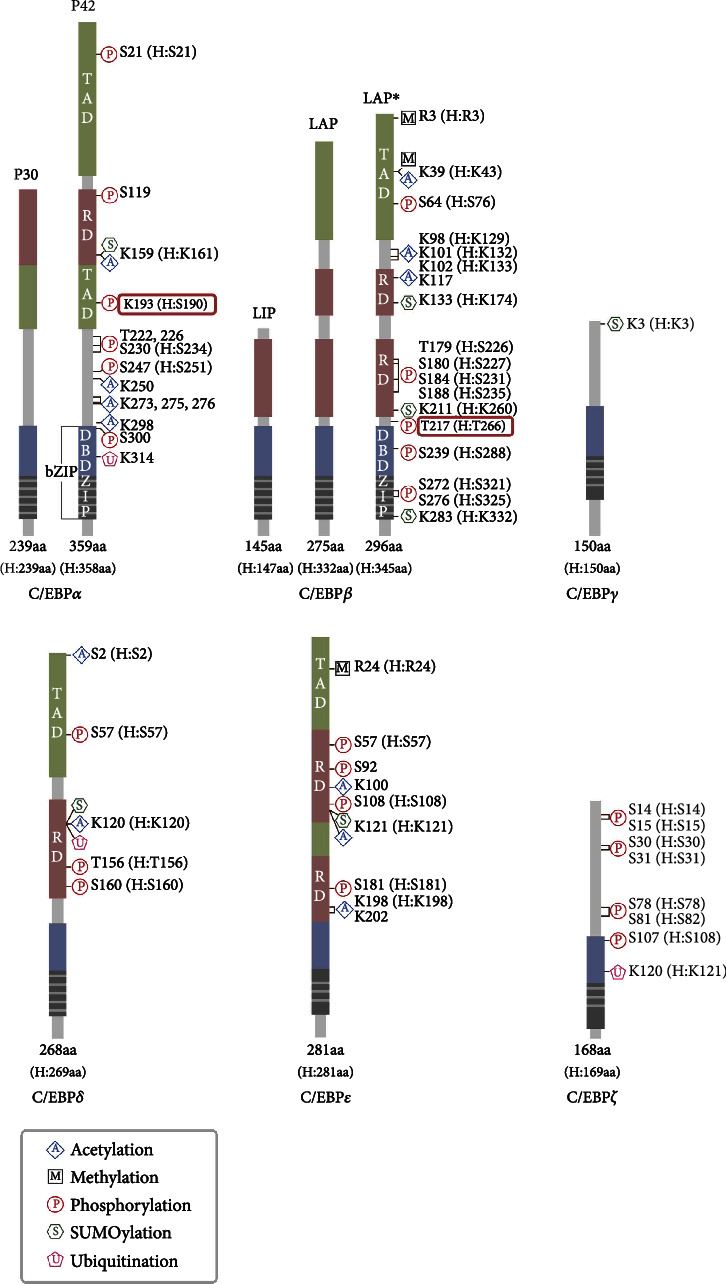
Posttranslational modifications of C/EBP family members. Two isoforms of C/EBP*α*, three isoforms of C/EBP*β*, and C/EBP*γ*, C/EBP*δ*, C/EBP*ε*, and C/EBP*ζ* are shown. The PTMs of C/EBPs mainly include phosphorylation, acetylation, ubiquitination, methylation, and SUMOylation. H in brackets is the abbreviation of human. K193-C/EBP*α* and T217-C/EBP*β* are important sites in fibrosis (DBD: DNA-binding domain; RD: regulatory domain; TAD: transcriptional activation domain; ZIP: leucine zipper).

C/EBP*α*, C/EBP*β*, and C/EBP*δ* are encoded by single-exon genes, C/EBP*γ* and C/EBP*ε* are encoded by genes with two exons, and *Cebpz* (*Ddit3*) contains four exons, two of which are within the 5′ untranslated regions (5′UTR). Athough there are only six genes, more than six C/EBP proteins can be present in tissues or cells. C/EBP*α* mRNA can produce two main polypeptides of 42 kDa (p42) and 30 kDa (p30), with the latter acting as an inhibitory isoform as it lacks the N-terminal transcriptional activation domain (TAD) [47]. Meanwhile, C/EBP*β* mRNA can give rise to at least three isoforms: 38 kDa (full liver activation protein (LAP^∗^)), 35 kDa (LAP), and 20 kDa (liver inhibitory protein (LIP)). LAP and LIP are the major C/EBP*β* forms in tissues and cells [48]. LAP contains both the activation and the bZIP domain, whereas LIP only possesses the bZIP domain and acts mainly as a negative inhibitor of C/EBPs by forming nontranscriptional active dimers with other C/EBP family members [48]. Some studies have reported that C/EBP*ε* mRNA can be translated into at least four isoforms: 32, 30, 27, and 14 kDa. Notably, the 30 kDa isoform has a lower activation potential than the 32 kDa isoform, while the 14 kDa isoform acts as the negative inhibitor as it lacks the intact N-terminal TAD [27, 49, 50]. Since the NCBI database only contains one section of the protein sequence, we present a 32 kDa protein of C/EBP*ε* containing 281 amino acids (aa) (Figures 1 and 2). On the molecular mechanism, different-sized C/EBP*α* and C/EBP*β* polypeptides can be produced by using alternative translation initiation codons in the same mRNA due to an upstream open reading frame (uORF) positioned in the 5′UTR [31]. Ribosomes scan the mRNA molecule from the 5′-cap and begin translation at the first AUG; however, this AUG is skipped when translating the uORF and translation begins at a downstream AUG [51]. Alternatively, the C/EBP*γ*, C/EBP*δ*, and C/EBP*ζ* mRNAs produce just one polypeptide.

### 2.2. C/EBP Proteins

The protein structure of C/EBPs has been studied extensively since the discovery of C/EBP*α* in the 1990s. All C/EBPs possess a highly conserved C-terminal (>90% sequence identity) containing a bZIP domain (Figure 2). The bZIP domain consists of a basic amino-acid-rich DNA-binding domain (DBD) and a leucine zipper (ZIP) dimerization domain that carries a heptad repeat of three (C/EBP*ε* and C/EBP*ζ*) or four (C/EBP*γ*) or five (C/EBP*α*, C/EBP*β*, and C/EBP*δ*) leucine residues that adopt an *α*-helical configuration [52]. Dimerization is a necessary for bZIP factors to bind to DNA via the DBD; however, it is now accepted that C/EBPs do not recognize the CCAAT box, but instead recognize 5′-(A/G)TT(G/A)CGAA(C/T)-3′ consensus DNA sequences [20, 31, 53–55]. The DBD also functions as the nuclear localization signal that mediates C/EBP translocation from the cytoplasm to the nucleus [56, 57]. Since the structure and DNA-binding characteristics of bZIP domains have been reviewed previously in excellent detail [29], we will not summarize these characteristics here.

The N-terminal region of C/EBPs is more varied than the C-terminal region. For instance, C/EBP*γ* and C/EBP*ζ* lack activation domains and instead repress gene transcription by building inactive heterodimers with other members. Meanwhile, C/EBP*α*, C/EBP*β*, C/EBP*δ*, and C/EBP*ε* carry both activation and regulatory domains, allowing them to serve as activators [21, 22, 31, 47, 48]. However, in some contexts, C/EBP*γ* and C/EBP*ζ* can also positively regulate transcription [58–60]. Besides, multiple C/EBP*α* and C/EBP*β* isoforms have been discovered (Figure 2) and have been reported to function as activators or inhibitors depending on the number of N-terminal activation domains [20].

In addition to dimerization with different C/EBP family members, C/EBPs can bind to other transcription factors and/or proteins in order to exert their functions. C/EBPs can not only dimerize with other bZIP transcription factors, such as Fos/Jun, cAMP response element-binding protein (CREB)/activating transcription factor (ATF) families, and/or AP1, but also interact with non-bZIP transcription factors including FOXOs, E2F, and NF-*κ*B [20]. Furthermore, various enzymes, such as kinases, acetylases, and enzymes, related to ubiquitination can bind to some C/EBPs and regulate their transcription function. For instance, C/EBP*α*, C/EBP*β*, C/EBP*δ*, C/EBP*ε*, and C/EBP*ζ* can be acetylated at different lysine residues to modulate their functions after binding to p300 [61–65]. Some C/EBP isoforms can also interact with other proteins and perform nontranscriptional functions. For example, C/EBP*δ* can bind to the Fanconi anemia group D2 protein (FANCD2) and facilitate its nuclear import [66]. Proteins that interact with C/EBPs and the effects of their interactions are summarized in Table S1.

### 2.3. C/EBP Posttranslational Modification (PTM)

PTM is crucial for many cellular biochemical and physiological activities in both mammals and plants [67–69]. Although hundreds of different types of PTM have been identified in eukaryotic proteomes, few have been studied extensively, including phosphorylation, acetylation, ubiquitination, glycosylation, methylation, small ubiquitin-like modifier modification (SUMOylation), and nitrosylation [70]. Before being translocated into the nucleus, C/EBPs undergo various PTM that can affect protein localization and stability, regulate DNA binding, and modulate interactions with transcription factors, cofactors, and other proteins [31]. Here, we mainly review C/EBP phosphorylation, acetylation, and SUMOylation, as well as ubiquitination and methylation (Figure 2).

Phosphorylation is the most common and well-studied PTM and is intimately involved in almost every cellular process. In particular, phosphorylation reversibly regulates protein activity through kinases and phosphatases [71, 72]. C/EBP*α* can be phosphorylated at S193 (serine at 193) by CDK4, which decreases its binding to C/EBP*β* and enhances complex formation with histone deacetylase 1 (HDAC1) or p300, thereby increasing the transcription of fatty acid synthesis-related genes and inhibiting cell cycle-associated gene expression [73–76]. The acetylation of lysine residues in nonhistone proteins plays a crucial role in many physiological functions, including protein folding and aggregation, RNA processing and stability, the cell cycle, and autophagy [70]. For example, the acetylation of C/EBP*ε* K121 (lysine at 121) and K198 enhances its DNA-binding activity during neutrophil differentiation [77]. The ubiquitination of lysine or methionine residues can also affect many proteasome-independent functions, especially proteasomal degradation [78]. Indeed, C/EBP*δ* K120 ubiquitination by siah E3 ubiquitin protein ligase 2 (SIAH2) can promote its proteasomal degradation [79]. Though a similar biochemical process to ubiquitination, the SUMOylation of lysine residue can modulate many protein functions, such as subcellular localization, protein-protein interactions, and protein-DNA binding [80]. C/EBP*δ* SUMOylation at K120 by SUMO1 abolishes its interaction with p300, thereby inhibiting *Cox-2* promoter activity [81]. The methylation of lysine or arginine residue plays an important role in protein stability, protein-protein interactions, protein-DNA interactions, and subcellular localization [82]. In C/EBP*β*, R3 (arginine at 3) methylation not only interferes with the recruitment of SWI/SNF-related, matrix-associated, actin-dependent regulator of chromatin, subfamily a, and member 4 (SMARCA4), but also regulates myeloid and adipogenic differentiation [83, 84]. Meanwhile, C/EBP*β* methylation at K39 inhibits the activation of myeloid genes and decreases its nuclear fraction [84, 85]. These C/EBP PTMs may play an important role in various diseases including fibrosis and be required to further study [19, 29, 31, 73].

### 2.4. C/EBP Location and Function

Although C/EBPs belong to the same family, their expression patterns can differ considerably. C/EBP*α* is highly expressed in numerous cell types, including adipocytes, hepatocytes, type II alveolar epithelial cells, and myeloid cells of the hematopoietic system [30], whereas C/EBP*β* has been detected in the heart, liver, adipose tissue, kidneys, intestine, and lungs [31, 86, 87]. C/EBP*γ* and C/EBP*ζ* are the ubiquitously expressed members of this family [25, 31]. Under normal physiological conditions, CEBP*δ* is expressed at low level in tissues and organs (except the liver, adipose tissue, intestine, lung, and brain) but can be rapidly induced by various events [31, 88]. C/EBP*ε* is exclusively expressed in myeloid cells of the bone marrow [89].

These differences in the expression of CEBPs indicate that they play important and unique roles in different tissues and organs. C/EBP*α* mainly serves as a transcription factor that modulates adipogenesis, lung development, hepatocyte lipid metabolism, myelopoiesis, and myeloid differentiation [30, 75, 90, 91]. Thus, gene mutations that result in C/EBP*α* protein dysfunction play vital roles in malignant myelopoiesis, especially in acute myeloid leukemia (AML) [92]. And C/EBPa knockout mice die shortly after birth due to impaired energy homeostasis [93]. Similarly, C/EBP*β* regulates the expression of genes involved in energy homeostasis and adipose tissue differentiation and affects endoplasmic reticulum (ER) stress and inflammation [94, 95]. Although some studies have reported that C/EBP*γ* exerts transactivation effects, it mainly functions as an inhibitor of C/EBPs and other interacted transcription factors [20, 25, 96]. Meanwhile, C/EBP*δ* transcriptionally modulates various biological processes such as cell differentiation, proliferation, motility, growth arrest, cell death, and inflammation depending on the cell type and cellular context [31, 32, 88]. Mice lacking C/EBP*δ* are viable, healthily, and exhibit no abnormalities, indicating that C/EBP*δ* is not vital for survival [53, 97]. In most cells, C/EBP*δ* expression is low under normal physiological conditions but can be rapidly induced by external stimuli [31]. Since C/EBP*δ* plays physiological roles in cell differentiation, proliferation, apoptosis, energy metabolism, and inflammation by regulating the expression of specific genes [31, 42], it may affect the pathogenesis of diseases such as fibrosis. Since C/EBP*ε* is mainly expressed in hematopoietic cells, it is essential for the late myeloid lineage differentiation and the functions of neutrophils and eosinophils [27, 98]. Indeed, C/EBP*ε* abnormalities are related to diseases such as neutrophil-specific granule deficiency (SGD), AML, and acute lymphoblastic leukemia [99–101]. Finally, C/EBP*ζ* has been reported to mediate ER stress-induced apoptosis [102] and regulate a wide range of genes involved in various cellular processes, such as inflammation, autophagy, and differentiation [21]. Thus, most C/EBP members can be detected in fibrosis-related cells such as fibroblasts, indicating that C/EBPs may play an important role in fibrosis.

## 3. Roles of C/EBPs in the Fibrotic Process

Increasing evidence has suggested that C/EBPs are closely associated with fibrogenesis [26, 40, 42, 90, 103, 104]. To date, several processes have been linked to the regulation of fibrosis by C/EBPs, including inflammation, lipid metabolism, cellular proliferation, apoptosis, autophagy, oxidative stress, ER stress, mitochondrial metabolism, macrophage polarization, and regulation of ECM gene expression. Here, we summarize the roles and mechanisms of C/EBPs in fibrosis.

It should be noted that there is no study about the role of C/EBP*ε* in fibrosis so far. Several existing studies have shown that mice lacking C/EBP*ε* display abnormal granulocyte terminal differentiation, decreased neutrophil infiltration into the lungs during ventilator-induced lung injury, and impaired phagokinetic motility of macrophages [105–107], indicating that C/EBP*ε* appears to participate in the onset and progression of multiple diseases by regulating the function of myeloid cells. Given the importance of myeloid cells in fibrosis-related diseases, further research should be required to elucidate the role of C/EBP*ε* in fibrotic diseases.

### 3.1. C/EBPs in Liver Fibrosis

**Figure 3:**
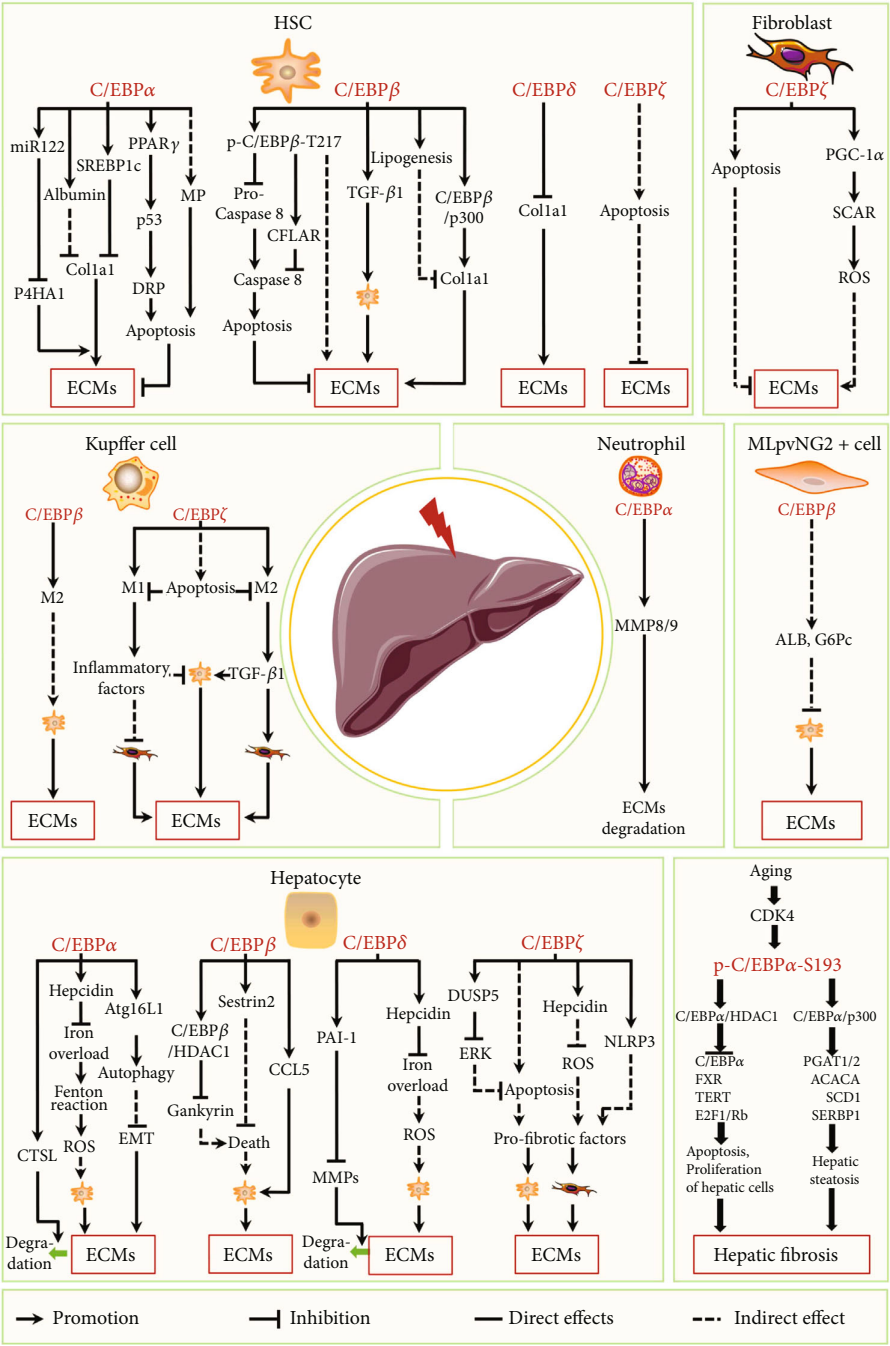
The mechanisms of C/EBPs in liver fibrosis. Hepatic stellate cells (HSCs), portal fibroblasts, hepatocytes, and immune cells such as Kupffer cells and neutrophils play vital roles in hepatic fibrosis. C/EBPs in these cells participate in the fibrotic process of the liver. C/EBP*α* in HSCs, hepatocytes, and neutrophils all exhibits antifibrotic effects on liver, but p-C/EBP*α*-S193 can promote hepatic fibrosis in aged liver through different mechanisms. C/EBP*β* and C/EBP*ζ* in different cells have different roles in hepatic fibrosis under various stimuli. C/EBP*δ* of HSCs and hepatocytes can inhibit liver fibrosis.

The liver is the critical hub of numerous physiological processes, including glucose and lipid metabolism [108, 109]; therefore, persistent liver dysfunction can affect the entire body and lead to diseases such as glycolipid metabolic disorder [109, 110]. Hepatic fibrosis, a pathophysiological result of two general types of chronic liver injuries: hepatotoxic injury and cholestatic injury, plays an important role in the development of various liver diseases through complex mechanisms [110, 111]. Fibrosis is the result of the interaction between varieties of cells. Myofibroblasts are the main source of ECM in hepatic fibrosis [14]. Hepatic stellate cells (HSCs) and portal fibroblasts are believed to be the major source of myofibroblasts in the fibrotic liver [112, 113]. In addition, it is now known that hepatocytes, macrophages, neutrophils, and mesenchymal stem cells also play the key roles in fibrosis [112]. This section review the roles of C/EBPs of these major cell types in liver fibrosis (Figure 3).

#### 3.1.1. C/EBP*α*

Endogenous C/EBP*α* expression is high in the normal liver tissues [73, 114] but is typically found at lower levels during liver fibrosis and under other pathological conditions [90, 105]. Treatment with carbon tetrachloride (CCl_4_, commonly used drugs to induce cirrhosis in animals) has been shown to cause hepatic fibrosis and decrease C/EBP*α* level in the livers of mice [28, 37, 115]. Furthermore, C/EBP*α* overexpression reduces CCl_4_-induced hepatic fibrosis in mice [37], indicating that C/EBP*α* plays a vital role in hepatic fibrosis.

In HSCs, high C/EBP*α* levels are essential for maintaining its quiescent state [116, 117]. Numerous studies have shown that C/EBP*α* expression decreases during HSC activation and that enhanced C/EBP*α* expression inhibits the HSC activation [28, 90, 118]. Furthermore, C/EBP*α* overexpression has been reported to suppress HSC activation by upregulating the expression of target genes, including *Albumin* and adipogenic transcriptional factors (peroxisome proliferator-activated receptor *γ* (*Pparγ*) and sterol regulatory element-binding protein 1c (*Srebp1c*)]) [116, 117, 119, 120]. Similarly, SREBP1c overexpression inhibits *α*1 (I) procollagen expression and causes a phenotypic reversal from activated to quiescent HSCs [120]. High C/EBP*α* levels also induce HSC apoptosis *in vitro* and *in vivo* via two pathways: (1) the mitochondrial pathway (MP) and (2) the death receptor pathway (DRP) regulated by PPAR*γ* and p53 [90, 121, 122]. In addition, C/EBP*α* has been reported to modify collagen maturation. *miR-122* is a target gene of C/EBP*α*, and the levels of both are decreased in activated HSCs [123]. miR-122 overexpression inhibits HSC proliferation and markedly attenuates prolyl-4-hydroxylase alpha polypeptide 1 (P4HA1) expression by targeting a binding site in 3′UTR of its gene, which hydroxylates the proline residue of collagen to allow its maturation [123]. C/EBP*α* overexpression also inhibits HSC proliferation by interacting with CDK2, CDK4, and E2F proteins [30, 118]. Together, this evidence indicates that C/EBP*α* levels correlate negatively with HSC activity and that C/EBP*α* upregulation could inhibit HSC activity and ECM production. Additionally, C/EBP*α* is involved in regulating HSC autophagy and a recent study showed that C/EBP*α* overexpression induces mitophagy in HSCs by binding to Beclin1 [124].

C/EBP*α* has been reported to function in terminally differentiated hepatocytes in the adult liver [90, 125]. C/EBP*α* overexpression inhibits hepatocyte proliferation by downregulating the expression of c-Myc and Cyclin D, reducing signal transducer and activator of transcription 3 (STAT3) phosphorylation, and improving liver function in a clinically relevant liver cirrhosis model [126, 127]. In addition, C/EBP*α* can regulate hepatic fibrosis through autophagy, with a recent study demonstrating that autophagy-related 16 like 1 (*Atg16L1*) is a target gene of C/EBP*α* in hepatocytes [128]. Atherogenic and high-fat diet-induced liver fibrosis mouse models display reduced C/EBP*α* and Atg16L1 expressions and increased liver fibrosis, while reversing high C/EBP*α* levels using peretinoin (an acyclic retinoid) increases autophagy activity [128]. A recent study showed that enhanced autophagy in the liver attenuates methionine-choline-deficient (MCD) diet-induced hepatic fibrosis and steatosis [129]. Conversely, inhibiting autophagy through hepatocyte-specific Atg5 or Atg7 deletion results in increased fibrosis in mouse livers [130, 131]. Furthermore, autophagy activation inhibits epithelial-mesenchymal transition (EMT) and hepatocyte differentiation into activated HSCs [132, 133]. During EMT, cells lose these epithelial characteristics, gain mesenchymal markers (e.g., vimentin, *α*-SMA, fibronectin, and fibroblast-specific protein 1), and express various collagens, resulting in increased ECM deposition [134].

C/EBP*α* also regulates iron metabolism in the liver to affect hepatic fibrosis. As one target of C/EBP*α*, hepcidin is thought to serve as a soluble modulator of iron metabolism by controlling intestinal iron absorption and iron release from macrophages [135]. Alcohol metabolism-mediated oxidative stress downregulates the expression and the DNA binding activity of C/EBP*α* in the liver, which reduces hepcidin levels in hepatocytes [136]. Low hepcidin levels cause iron overload in hepatocytes, which can increase the Fenton reaction to generate abundant reactive oxygen species (ROS) that cause grave cellular and tissue damage, thereby contributing toward fibrosis [137]. Besides, C/EBP*α* also regulates hepatic fibrosis by affecting the synthase and secretion of matrix-degrading proteases in hepatocytes. Cathepsin L (CTSL), a target of C/EBP*α*, is an extracellular matrix-degrading protease secreted by hepatocytes whose expression in hepatic cell lines is downregulated by acetaldehyde, an oxidative metabolite of ethanol [138]. In addition, decreased CTSL expression may partly contribute toward ECM deposition in alcoholic liver fibrosis [138, 139].

C/EBP*α* regulates the progression of hepatic fibrosis by modifying the secretory functions of neutrophils. For instance, liver fibrosis is alleviated and neutrophil numbers are increased in the liver of Tribbles pseudokinase 1- (Trib1-) deficient mice treated with CCl_4_ [24]. Trib1 plays an essential role in C/EBP*α* degradation by recruiting it to E3 ubiquitin ligase [24, 140], with C/EBP*α* expression reported to be upregulated in neutrophils in Trib1-deficient mice [141]. Further research has shown that C/EBP*α* overexpression directly induces matrix metalloproteinase- (MMP-) 8 and MMP-9 expressions and secretion from neutrophils, thereby mediating ECM degradation in the liver [24].

C/EBP*α* phosphorylation ar Ser193 in mice (p-C/EBP*α*-S193, S190 in human) plays an essential role in age-associated hepatic fibrosis. Aged livers are more susceptible to pharmacological therapies and are more likely to develop liver diseases such steatosis, cirrhosis, and fibrosis, at least partly due to increased p-C/EBP*α*-S193 caused by increased CyclinD3/CDK4 activity [73, 75, 142]. In aged livers, total C/EBP*α* protein levels are descended and p-C/EBP*α*-S193 levels are increased [73, 143]. P-C/EBP*α*-S193 upregulation in liver cells increases the formation of C/EBP*α*-p300 complex, which directly activates the promoters of triglyceride synthesis-related genes (diacylglycerol O-acyltransferase 1/2 (*Dgat1/2*), acetyl-coenzyme A (*Acaca*), stearoyl coenzyme A desaturase 1 (*Scd1*), and *Srebp1*), resulting in hepatic steatosis and fibrosis [75, 142, 143]. In addition, increased p-C/EBP*α*-S193 augments the C/EBP*α*-HDAC1 complex in aged liver under CCl_4_ treatment, which directly represses the promoters of *Cebpα*, Farnesoid X receptor (*Fxr*), and telomere reverse transcriptase (*Tert*) genes and disrupts the Rb-E2F1 complex, leading to increased apoptosis, accelerated liver cell proliferation (including HSCs), and the early development of liver fibrosis [73].

In summary, C/EBP*α* can negatively regulate liver fibrosis by regulating the functions of HSCs, hepatocytes, and other cells through various mechanisms. Notably, p-C/EBP*α*-S193 positively regulates age-associated hepatic fibrosis by affecting the formation and function of C/EBP*α*-p300 and C/EBP*α*-HDAC1 complexes.

#### 3.1.2. C/EBP*β*

C/EBP*β* is detected in normal liver tissue but its protein levels or transcriptional activity decrease during various hepatic disease, including thioacetamide (a drug widely used for induction of fibrosis and acute liver failure)- or dimethylnitrosamine (DEN, a drug used to induce liver cirrhosis in experimental animals)-induced liver fibrosis, methionine-, and choline-deficient diet (MCD, used to induce nonalcoholic fatty liver disease)-induced liver fibrosis, CCl_4_-induced liver fibrosis, and streptozotocin (a drug can damage pancreatic beta insular cells)-induced diabetes [103, 144–147]. C/EBP*β* deficiency has been shown to inhibit CCl_4_-induced liver fibrosis [148]; however, other reports have shown that C/EBP*β* protein levels or DNA binding activity increase in models of liver fibrosis induced by CCl_4_ [73, 149, 150]. Thus, C/EBP*β* may play different roles in hepatic fibrosis.

HSCs are modulated by C/EBP*β* activity [150]. One point is that high C/EBP*β* levels or activity promotes HSC activation. C/EBP*β* interacts with p300 to bind to the *Col1α1* promoter and enhance its expression in HSCs induced by TGF-*β*1 or acetaldehyde [148, 151, 152]. Col1*α*1 stimulates further HSC activation and increases the phosphorylation-mediated activation of C/EBP*β*-Thr217 (p-C/EBP*β*-T217; Thr266 in human) [149, 153]. In addition, CCl_4_ upregulates p-C/EBP*β*-T217 through increasing ribosomal S6 kinase (RSK), which inhibits caspase 8 (Casp 8) activation not only by interacting with p-C/EBP*β*-T217 and proCasp8 but also by increasing the expression of Casp 8 and Fas-associated via death domain-like apoptosis regulator (CFLAR), a critical Casp 8 inhibitor, leading to decreased HSC apoptosis [149, 150, 153]. High p-C/EBP*β*-T217 levels also promote HSC proliferation and activation, resulting in ECM production [150]. Alternatively, it has been reported that C/EBP*β* negatively regulates HSC activation. When HSCs differentiate from quiescent lipid-storing cells into activated myofibroblasts to participate in liver fibrogenesis, C/EBP*β* protein levels decrease [120]. It has shown that C/EBP*β* can bind to the *Tgf-b1* promoter and negatively regulate its expression [103]. Under DEN administration, the nuclear translocation of cytosolic C/EBP*β* induced by oltipraz (5-(2-pyrazinyl)-4-methyl-1,2-dithiol-3-thione) inhibits TGF-*β*1 expression in HSCs, resulting in HSC inactivation and reduced ECM accumulation [103].

Hepatocyte C/EBP*β* also plays different roles in liver fibrosis. ER stress increases C/EBP*β* level in hepatocytes, which directly upregulates the expression of Sestrin2, a conserved antioxidant protein, thus resisting hepatocyte death [154]. A recent study showed that C/EBP*β* levels decrease in hepatocytes induced by low FXR under DEN treatment, resulting in decreased C/EBP*β*-HDAC1 complex formation [155]. This complex directly removes Gankyrin inhibition, leading to hepatocyte death through the Rac1/JNK pathway [155, 156]. Hepatocyte death is a key trigger for liver fibrosis progression; however, high C/EBP*β* levels in hepatocytes can promote HSC activation by the C-C chemokine ligand 5 (CCL5) /CCR5 pathway. Lipid accumulation in hepatocytes also upregulates C/EBP*β*, which binds to the *Ccl5* promoter to increase its expression and secretion [157]. CCL5 subsequently binds to its receptor CCR5 in HSCs to boost their proliferation and collagen production [157].

Liver fibrosis also involves C/EBP*β* expression in other nonparenchymal cells including Kupffer cells (the macrophages in liver). For instance, C/EBP*β* levels are increased in miR-155 (its target gene *Cebpb*)-deficient Kupffer induced by lipopolysaccharide (LPS), resulting in M2 polarization and subsequent HSC activation through various mechanisms [158]. Besides, in the DEN-induced liver fibrotic/cirrhotic mouse model, increased C/EBP*β* levels induce hepatic-sourced mesenchymal stem cell (MLpvNG2^+^) differentiation into hepatocytes (ALB^+^G6Pc^+^), thereby alleviating liver injury and inhibiting HSC activation [159].

In short, the roles of C/EBP*β* in various cell types are different in the fibrosis process. Even in the same cell type such as HSCs, C/EBP*β* effects on fibrosis are different under various stimuli. The possible reason is that the *Cebpb* gene can encode at least three isoforms with different functions in these cells. Future investigations should explore the roles of different C/EBP*β* isoforms in various cell types during liver fibrosis.

#### 3.1.3. C/EBP*δ*

Stimuli such as LPS, sepsis, endotoxemia, and partial hepatectomy can induce C/EBP*δ* expression in the liver tissue, indicating that C/EBP*δ* is involved in the hepatic diseases, including fibrosis [28, 160, 161]. It has been suggested that C/EBP*δ* may inhibit HSC activation, since C/EBP*δ* protein levels are decreased in activated HSCs but increased in quiescent HSCs [120]. Furthermore, treating HSCs with LPS or TNF-*α* can upregulate C/EBP*δ*, which binds to the *Col1a1* promoter to inhibit its expression [28, 162]. Together, these studies illustrate that C/EBP*δ* plays an important role in limiting hepatic fibrosis via inhibiting HSC activation.

In hepatocytes, C/EBP*δ* expression can be stimulated by IL-1*β* and IL-6 [163, 164], and high C/EBP*δ* levels are maintained by autoregulation mechanisms through binding to the C/EBP sites located downstream of the *Cebpd* gene in HpeG_2_ cells [165]. In addition, IL-1*β* can elevate C/EBP*δ* levels and activate STAT3 in hepatocytes, which together upregulate hepcidin expression [163, 166]. High levels of hepcidin can prevent iron overload in hepatocytes, thereby reducing the development of hepatic fibrosis [137]. However, IL-6-stimulated C/EBP*δ* can be mediated by STAT3 and SP1 to bind to the plasminogen activator inhibitor-1 (*Pai-1*) promoter and upregulate its expression [164, 167]. Elevated PAI-1 levels inhibit plasmin-dependent MMP activity, thereby contributing toward the excessive accumulation of collagen and other ECM protein in tissue [168]. These data indicate that, although inflammatory cytokines can stimulate C/EBP*δ* expression, different inflammatory factors regulate different gene expressions through upregulating C/EBP*δ*, which can inhibit or promote liver fibrosis.

#### 3.1.4. C/EBP*ζ*

C/EBP*ζ* was first identified during ultraviolet irradiation research [169]. Like C/EBP*δ*, C/EBP*ζ* is extremely weakly expressed under normal physiology but is highly expressed under cellular stress [21]. Numerous studies have confirmed that C/EBP*ζ* is the main executor of ER stress-induced apoptosis and regulates numerous genes involved in autophagy, differentiation, and inflammation [21, 117]. It has been suggested that C/EBP*ζ* promotes fibrogenesis in the liver, as indicated by reduced hepatic fibrosis in mice lacking C/EBP*ζ* under various stimuli [40, 170–172]. However, some reports have shown that C/EBP*ζ* exerts antifibrotic effects on the liver *in vivo* and *in vitro* [41, 173]. This may be due to the different responses of C/EBP*ζ* in different liver cells under different stimuli.

The role of C/EBP*ζ* in ECM-producing liver cells, including HSCs and liver fibroblasts, remains somewhat controversial. C/EBP*ζ* upregulation caused by the deletion of heat shock protein 47 under autophagy inhibition or stimulation by cannabidiol can induce HSC apoptosis [174, 175]. Similarly, C/EBP*ζ* upregulation induced by the accumulation of matricellular proteins can induce apoptosis in myofibroblasts derived from liver fibroblasts, which mitigates liver fibrogenesis by decreasing the cellular productions of fibronectin, Col1a1, and *α*-SMA [173]. However, increased C/EBP*ζ* levels are observed in activated HSCs by cultured or treated with ER stress inducer, suggesting that C/EBP*ζ* may play a role in HSC activation rather than apoptosis [176]. In addition, the upregulation of C/EBP*ζ* induced by free fatty acids in liver fibroblasts directly inhibits *Pgc-1a* transcription and protein expression, which impairs mitochondrial steatohepatitis-associated circRNA ATP5B regulator (SCAR) expression, leading to ROS production and fibroblast activation [177].

Hepatocyte C/EBP*ζ* promotes liver fibrosis. As a major player of ER stress, the upregulation of C/EBP*ζ* induced by multiple stimuli can lead to hepatocyte apoptosis [178–180]. Apoptosis is a frequent cellular process that causes organ remodeling and fibrosis in response to injury [21]. C/EBP*ζ* directly activates apoptotic pathways by altering the transcription of proapoptotic or antiapoptotic genes and can directly bind to the dual-specificity phosphatase 5 (*Dusp5*) promoter and upregulate its expression, thereby decreasing ERK activity and leading to hepatocyte apoptosis under CCl_4_ treatment [178]. In addition, C/EBP*ζ* upregulation induced by ER stress can directly increase the protein levels of NACHT, LRR, and PYD domains-containing proteins 3 (NLRP3), which can mediate inflammasome activation in hepatocytes, aggravating liver injury and fibrosis [181]. C/EBP*ζ* can also induce hepcidin expression by inhibiting C/EBP*α* activation in hepatocytes treated with thioacetamide, resulting in a significant hepatic iron overload [182]. Alongside inflammasome activation, iron overload can aggravate hepatocyte damage, which in turn promotes liver fibrogenesis [137, 183].

The role of macrophages C/EBP*ζ* in liver fibrosis remains inconsistent. For instance, high C/EBP*ζ* levels are observed in F4/80^+^CD206^+^ macrophages (M2) during schistosomiasis-induced liver fibrogenesis [184] and C/EBP*ζ* overexpression has been reported to stimulate M2 polarization through the KLF4/STAT6 pathway [185]. These data indicate that M2 macrophages induced by C/EBP*ζ* play an important role in schistosomiasis-induced liver fibrogenesis. However, a recent report shows that C/EBP*ζ* knockdown restores Arg1 and Mrc1 expressions, increases STAT3 and STAT6 activation, and enhances IL-10 secretion in Kupffer cells under hyperglycemic conditions, indicating that C/EBP*ζ* is involved in high glucose-induced M1 macrophage polarization [186]. In addition, C/EBP*ζ*-deficient bone marrow-derived macrophages resist apoptosis when treated with palmitic acid, leading to the accumulation of activated macrophages in the liver and subsequent liver fibrosis. Thus, macrophage C/EBP*ζ* may protect the liver from fibrogenesis by limiting macrophage survival during lipotoxicity [41].

In summary, these existing studies about C/EBP*ζ* in liver fibrosis show that C/EBP*ζ* overexpression in profibrotic cells such as HSCs, fibroblasts, and M2 macrophages can lead to their apoptosis, inhibiting liver fibrosis, while its overexpression in nonprofibrotic cells such as hepatocytes and M1 macrophages can result in their apoptosis, promoting liver fibrosis by activating profibrotic cells.

### 3.2. C/EBPs in Lung Fibrosis

**Figure 4:**
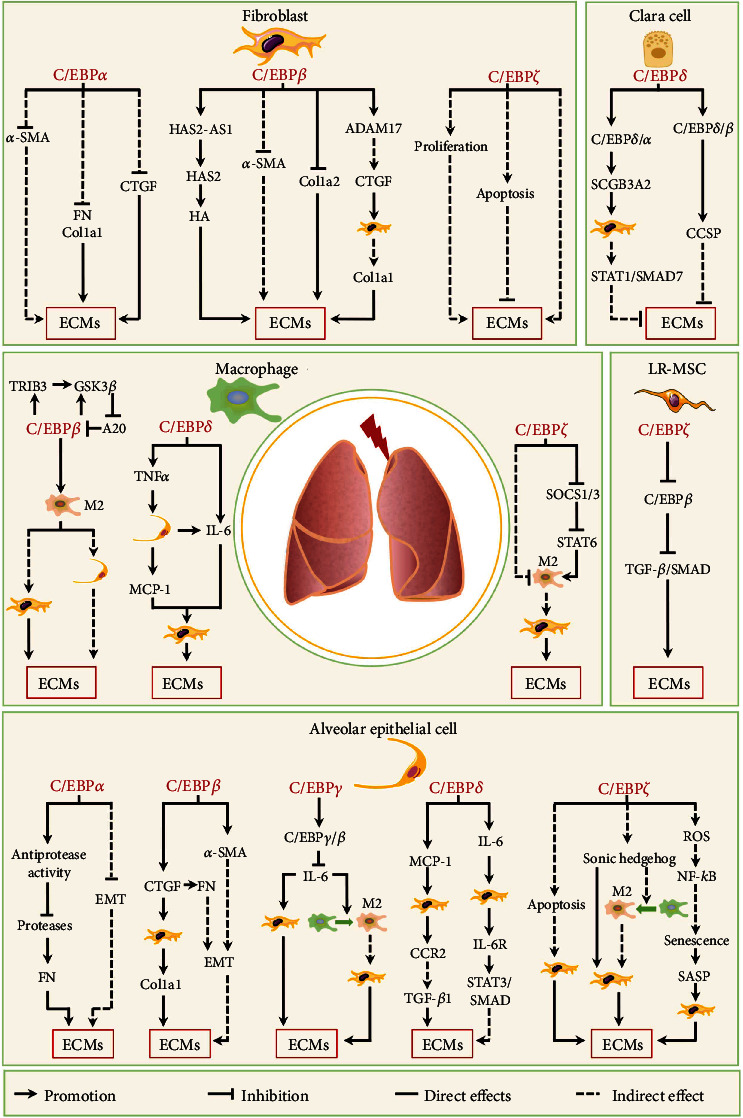
The mechanisms of C/EBPs in lung fibrosis. C/EBP*α* proteins in both lung fibroblasts and alveolar epithelial cells all exert inhibitory effects on lung fibrosis. C/EBP*β* in different cells have different roles in pulmonary fibrosis under various stimuli. C/EBP*γ* of alveolar epithelial cells can inhibit lung fibrosis. C/EBP*δ* in lung macrophages and alveolar epithelial cells possess promotive effects on pulmonary fibrosis whereas Clara cell C/EBP*δ* inhibits lung fibrosis. In addition to its diverse roles in macrophages, C/EBP*ζ* in other cells can promote pulmonary fibrosis under various stimuli.

Lung fibrosis is a pathological process associated with various respiratory diseases, including immunological diseases (scleroderma and sarcoidosis), infection, and lung injury caused by chemicals, radiation, and environmental exposures [2, 112]. Pulmonary fibrosis is characterized by ECM deposition in interstitial and reduced lung compliance, and restrictive lung function and progressive lung fibrosis can lead to lung hypertension, right heart failure, and ultimately respiratory failure [2]. In addition to common molecular signaling pathways such as TGF-*β*, CTGF, Hedgehog, Notch, and fibroblast growth factor (FGF) [187], recent studies have shown that fibroblasts, alveolar epithelial cells, macrophages, Clara cells, and lung resident mesenchymal stem cell (LR-MSC) can been implicated in lung fibrosis through various mechanisms [14, 52, 188–191]. Here, we discuss the roles of C/EBPs of these cell types in lung fibrosis (Figure 4).

#### 3.2.1. C/EBP*α*

C/EBP*α* is expressed in various lung cells types, including alveolar type II cells, lung fibroblasts, alveolar macrophages, Clara cells, and bronchial smooth muscle cells [43, 91, 192, 193]. Under physiological conditions, C/EBP*α* regulates lung development and maturation [194, 195]; however, C/EBP*α* expression is markedly decreased in diseased lung or those subjected to harmful stimuli, indicating that C/EBP*α* plays an important role in the progress of pulmonary diseases, such as lung fibrosis [192, 196–198].

Lung fibroblasts regulate tissue homeostasis and the balance between tissue repair and fibrosis. C/EBP*α* mRNA and protein levels are significantly decreased in fibroblasts isolated from the lung tissue of patients with idiopathic pulmonary fibrosis (IPF, a chronic progressive fibrotic disease) [43]. Furthermore, the siRNA-mediated loss of C/EBP*α* in normal lung fibroblasts enhances the profibrotic activation and ECM deposition, whereas C/EBP*α* upregulation by transient transfection in IPF-derived fibroblasts significantly reduces profibrotic genes expression and ECM production and while promoting lipid droplet formation [43]. Thus, C/EBP*α* could promote the dedifferentiation of myofibroblasts to fibroblasts to inhibit the lung fibrosis.

Recent evidence has suggested that the alveolar epithelium plays a central role in the pathogenesis of lung fibrosis [199]. Since C/EBP*α* has been detected in alveolar epithelial cells, C/EBP*α* may regulate the function of alveolar epithelial cells to affect lung fibrosis. However, the role of alveolar epithelial cell C/EBP*α* in pulmonary fibrosis is somewhat controversial. Didon et al. reported that epithelial-specific C/EBP*α* disruption results in the lung interstitial fibrosis, indicating that C/EBP*α* levels in alveolar epithelial cells correlate negatively with fibrosis [196]. Although no studies have examined the underlying mechanism, the disrupted dispersion of airway smooth muscle cells suggests that epithelial cells can transdifferentiate to mesenchymal cells [196]. However, Sato et al. have shown that conditional C/EBP*α* deletion in alveolar type II cells and Clara cells leads to the decreased fibronectin deposition [200]. In particular, C/EBP*α* regulates the protease/antiprotease balance by increasing the expression and activity of antiprotease to inhibit the protease activity in the lungs, which suppresses fibronectin degradation and lung fibrosis during the repair process [200], possibly due to the different effects of C/EBP*α* knockout in the lung tissue. Didon et al. constructed C/EBP*α*-knockout mice with *Cebpa*^fl/fl^ mice and *Spc-Cre*^+^ mice (surfactant protein C (SP-C) promoter active in all lung epithelial cells), in which the C/EBP*α* was deleted in all SP-C-expressed epithelial cells [196]. Sato et al. deleted *Cebpa* gene in the lungs using *Scgb1a1-rtTA*^-/tg^/*(tetO)_7_CMV-Cre*^tg/tg^/*Cebpa*^fl/fl^ mice [200]. SCGB1A1 (secretoglobin family 1A member 1) is primarily expressed and secreted by the Clara cells [201]. Although C/EBP*α* was deleted in alveolar type II cells of these mice, its effect on positive regulation of lung fibrosis may be mainly mediated through Clara cells. Thus, further research is required to determine the precise function of C/EBP*α* in different cells, including Clara cells, during pulmonary fibrosis.

In brief, existing evidence shows that C/EBP*α* upregulation inhibits fibroblast activation by restraining *α*-SMA, FN, Col1a1, and CTGF expressions and suppresses the ECM production in alveolar epithelial cells through inhibiting its EMT and promoting the activity of antiprotease alveolar epithelial cells, indicating that targeting C/EBP*α* may be an effective strategy to treat pulmonary fibrosis.

#### 3.2.2. C/EBP*β*

In the lungs, C/EBP*β* is expressed in parenchymal, mesenchymal, and infiltrated inflammatory/immune cells [42, 192, 202]. C/EBP*β* protein levels or transcriptional activity are increased in lung tissues under various profibrotic stimuli, with C/EBP*β* knockout inhibiting pulmonary fibrosis [39, 42, 190, 192]. Therefore, C/EBP*β* may play an important role in lung fibrosis.

As a crucial fibrogenic factor, TGF-*β*1 can upregulate C/EBP*β* protein levels in primary lung fibroblasts *in vitro* [203]. Compared to lung fibroblasts isolated from wild-type mice treated with bleomycin (common drug for inducing pulmonary fibrosis), lung fibroblasts isolated from bleomycin-treated C/EBP*β* null mice exhibit lower *α*-SMA levels and greater proliferation ability [39]. Together, these studies indicate that C/EBP*β* may regulate lung fibroblast activation. As a mediator of lung fibrosis activation, hypoxia can promote C/EBP*β* phosphorylation at Thr266 (Thr217 in mice), which enhances its binding to a disintegrin and metalloproteinase 17 (*ADAM17*) promoter and ultimately induces ADAM17 expression in human lung fibroblasts [204]. ADAM17 overexpression in lung fibroblasts affects the hypoxia-induced expression of CTGF [204], which can induce Col1a1 and *α*-SMA expression by activating the Rac1/MLK3/JNK/AP-1 pathway [205]. Besides, hypoxia-induced C/EBP*β* expression can upregulate antisense of hyaluronan synthase 2 (HAS2-AS1) in lung fibroblasts, which promotes HAS2 expression though HAS2 mRNA/HAS2-AS1 heterodimer formation [206, 207]. HAS2 is an enzyme responsible for the synthesis of the ECM component hyaluronan (also known as hyaluronate or hyaluronic acid (HA)). The overexpression of HAS2 and HA has been implicated in severe fibrosis [208]. In addition, the key inflammatory cytokine TNF-*α* can inhibit Col1a2 transcription in cultured fibroblasts by stimulating C/EBP*β* protein expression [104]. However, C/EBP*β* protein level is also induced by IL-1*β* in lung fibroblasts, with a greater increase in C/EBP*β*-LIP isoform expression leading to a reduced LAP/LIP ratio and reduced *α*-SMA promoter activity and expression [209]. This may explain why treatment with inflammatory factors increases C/EBP*β* expression and decreases fibroblast activation [210, 211].

In the alveolar epithelial cells, *α-Sma* and *Ctgf* are target genes of C/EBP*β* [212, 213]. As an important factor in EMT, TGF-*β*1 increases C/EBP*β* levels in alveolar epithelial cells and allows C/EBP*β* to bind to *α-Sma* and *Ctgf* promoters and increase their protein levels [212, 213]. CTGF overexpression induces fibronectin expression in alveolar epithelial cells and increased *α*-SMA and fibronectin levels active the EMT, resulting in ECM deposition [212]. In addition, CTGF secreted from alveolar epithelial cells can activate the fibroblasts to produce ECM in a paracrine manner [205]. Thus, C/EBP*β* may positively regulate the occurrence and development of pulmonary fibrosis in alveolar epithelial cells.

C/EBP*β* expression in monocytes/macrophages is also involved in lung fibrosis. Recent studies have shown that C/EBP*β* deficiency results in a complete lack of segregated nucleus-containing atypical monocytes (Ly6C^−^F4/80^−^Mac1^+^Ceacam1^+^Msr1^+^) derived from Ly6C^−^Fc*ε*RI^+^ granulocyte/macrophage progenitors, preventing the development of bleomycin-induced lung fibrosis [190]. Moreover, high C/EBP*β* levels in monocyte-derived alveolar macrophages, but not tissue-resident alveolar macrophages, promotes lung fibrosis [42, 214]. During lung injury, the stress response protein Trib3 in monocyte-derived alveolar macrophages interacts with GSK-3*β* and protects it from ubiquitination and degradation [214]. GSK-3*β* phosphorylates ubiquitin-editing enzyme A20 to inhibit its ubiquitin-editing activity, causing C/EBP*β* accumulation in macrophages [42]. Activated C/EBP*β* not only directly increases the transcription of *Trib3* and *Gsk-3b*, thereby establishing a positive feedback loop in macrophages, but also enhances the expression of its targeted genes (*Arg1*, *Il10*, *Tgfb1*, and *Fizz1*) to promote a profibrotic macrophages (M2) phenotype and lung fibrosis [42, 214, 215].

In summary, these studies show that C/EBP*β* upregulations of different cell types exert the profibrotic effect on pulmonary fibrosis. Given that LAP isoform is detected in most of these studies, C/EBP*β* may play the profibrotic role through LAP isoform. Further research may be required to investigate the role of C/EBP*β*-LIP isoform in the profibrotic effect of C/EBP*β* in lung fibrosis.

#### 3.2.3. C/EBP*γ*

C/EBP*γ* can only form stable heterodimerizes with other transcription factors, including C/EBPs, thereby regulating their transcriptional activities [216–218]. Although C/EBP*γ* is involved in various cellular processes, such as the integrated stress response, cell proliferation, senescence, natural killer cell maturation, and glucose utilization [219–222], there is no report about the role of C/EBP*γ* in the fibrogenesis. And a few studies have also focused on the role of C/EBP*γ* in regulating lung inflammation and wound repair. Since inflammation plays an important role in fibrosis, we will review the regulatory role of C/EBP*γ* in lung inflammation.

C/EBP*γ* can improve lung inflammation caused by a pathogenic stimuli, indicating that it may alleviate lung fibrosis. During acute lung injury induced by LPS and IgG immune complex, C/EBP*γ* overexpression in lung tissue alleviates pulmonary damage by reducing vascular permeability changes, decreasing the recruitment of neutrophils into alveolar spaces, and inhibiting the production of inflammatory mediators [223]. Mechanistically, C/EBP*γ* overexpression inhibits inflammation by reducing the transcription activities of C/EBP*β* and C/EBP*δ* [223]. IL-1*β* induces C/EBP*γ* activation in lung epithelial cells, which attenuates the transcription activity of C/EBP*β* and inhibits IL-6 expression [25]. IL-6 boosts lung fibrosis by not only activating pulmonary fibroblasts but also promoting M2 macrophage polarization [39, 224]. These reports indicate that C/EBP*γ* indirectly suppresses lung fibrosis through inhibiting inflammation.

#### 3.2.4. C/EBP*δ*

In the lung, C/EBP*δ* is detected in alveolar type II cells, alveolar macrophages, Clara cells, and bronchial smooth muscle cells under physiological conditions [44, 225–227]. C/EBP*δ* expression can be induced in lung tissue by multiple stimuli, including bacterial infections and LPS stimulation [33, 228–230]; however, it remains unclear whether C/EBP*δ* directly regulates pulmonary fibrosis. Here, we discuss recent studies on the effect of C/EBP*δ* in alveolar type II cells, alveolar macrophages, Clara cells, and fibroblasts to illustrate its role in lung fibrosis directly or indirectly.

As a direct upstream transcription factor of IL-6 and TNF-*α*, C/EBP*δ* mediates the stimulation of these cytokines by LPS in macrophages during acute lung injury [44, 223]. In addition, inflammatory factors including TNF-*α* and IL-1*β* can induce the expression of C/EBP*δ*, which binds to the IL-6 and MCP-1 promoters and augments their expressions in alveolar type II cells [225, 231]. Various studies have shown that these cytokines participate in the regulation of pulmonary fibrosis. For instance, IL-6, a soluble mediator with a pleiotropic effects, can activate the STAT3/SMAD signaling pathway by binding to IL-6R in order to induce ECM expression in lung fibroblasts, thereby exacerbating bleomycin-induced lung fibrosis [224, 232, 233]. In addition to its role in monocyte recruitment to sites of inflammation, MCP-1 can stimulate ECM expression in lung fibroblasts by binding to its receptor CCR2 and endogenous upregulating TGF-*β*1 expression [234, 235]. Thus, C/EBP*δ* may accelerate lung fibrosis through indirectly inducing inflammatory cytokines in pulmonary epithelial cells and alveolar macrophages.

In Clara cells, C/EBP*δ* forms heterodimers with C/EBP*α* that can not only bind to C/EBP-response element sites in the Clara cell secretory protein (*Ccsp*) promoter to activate its expression but also enhance secretoglobin 3A2 (SCGB3A2) expression [236, 237]. In addition, C/EBP*δ* dimerizes with C/EBP*β* to induce CCSP expression in Clara cells under glucocorticoid stimulation [238]. CCSP deficiency in obliterative bronchiolitis results in greater lung injury and fibrosis, indicating that CCSP inhibits lung fibrosis [239]. Meanwhile, SCGB3A2 exerts antifibrotic effects in bleomycin-induced pulmonary fibrosis by inhibiting TGF-*β*1-induced fibroblast activation via increased STAT1 phosphorylation and SMAD7 expression, and decreased SMAD2 and SMAD3 phosphorylation [240, 241]. These findings suggest that C/EBP*δ* can inhibit pulmonary fibrosis through upregulating secretory proteins in Clara cells.

Therefore, C/EBP*δ* upregulation in alveolar epithelial cells and macrophages can promote lung fibrosis through activating fibroblasts. However, C/EBP*δ* upregulation in terminal bronchiole cells (Clara cell) could inhibit lung fibrosis by different mechanisms. These findings indicate that C/EBP*δ* in different regions of lung tissue could play different roles in fibrosis process.

#### 3.2.5. C/EBP*ζ*

C/EBP*ζ* expression is increased in the lung tissues of patients with IPF [45, 242] and can be upregulated by multiple stimuli, including bleomycin, hypoxia, and silica, to induce lung fibrosis [45, 243, 244]. Although these studies suggest that C/EBP*ζ* may play an important role in the occurrence and development of pulmonary fibrosis, growing evidence has shown that C/EBP*ζ* does not exert the same effect on pulmonary fibrosis in different lung cells.

In the lung tissues of IPF and pulmonary fibrosis models, C/EBP*ζ* is mainly located in alveolar epithelial cells [242, 245]. ER stress caused by multiple stimuli such as hypoxia and increased hypoxia inducible factor-1*α* (HIF-1*α*) can upregulate C/EBP*ζ* in alveolar epithelial cells, which subsequently promotes the expression of proapoptotic genes and inhibits antiapoptotic genes, leading to apoptosis and potential organ remodeling and fibrosis [21, 45, 242, 243]. In addition, ER stress-induced C/EBP*ζ* upregulation can increase Sonic Hedgehog expression and promote its secretion from type II alveolar epithelial cells, which then activates lung fibroblasts through activating the Hedgehog signaling pathway and polarizes macrophages into the M2 stage in an osteopontin-dependent manner, resulting in pulmonary fibrosis [246, 247]. Furthermore, increased C/EBP*ζ* levels can exacerbate lung fibrosis by inducing senescence in alveolar epithelial cells [248]. *In vivo* and *in vitro* studies of bleomycin and tunicamycin (a drug is employed to induce ER stress) have shown that C/EBP*ζ* upregulation can induce alveolar epithelial cell senescence through the ROS/NF-*κ*B pathway, which activates lung fibroblasts mediated by the senescence-associated secretory phenotype, promoting a pulmonary fibrosis pathology [248, 249].

C/EBP*ζ* is mainly involved in the activation of lung fibroblasts. Thrombin, SiO_2_, and bleomycin can induce the ER stress via the PI3K/AKT/mTOR pathway to stimulate the upregulation of C/EBP*ζ* protein, which activates lung fibroblasts [244, 245, 250]. Increased C/EBP*ζ* levels also induce lung fibroblast apoptosis, but to a lesser degree than their proliferation, resulting in increased cell numbers [244, 251]. In addition, lung resident mesenchymal stem cells (LR-MSCs) can transform to myofibroblast to promote lung fibrogenesis [252]. C/EBP*ζ* overexpression caused by ER stress or exogenous DNA facilitates transformation of LR-MSC into myofibroblast induced by TGF-*β*1, which binds C/EBP*β* to eliminate TGF-*β*/SMAD signaling pathway-mediated inhibition [253].

Despite these findings, the role of macrophage C/EBP*ζ* in pulmonary fibrosis remains unclear. Although C/EBP*ζ* is primarily detected in alveolar epithelial cells under fibrotic stimuli, it is also expressed in lung macrophages [254]. Bleomycin-induced pulmonary fibrosis is found to be significantly attenuated in C/EBP*ζ*-deficient (C/EBP*ζ*^−/−^) mice; however, the number and polarization of alveolar and interstitial macrophages does not differ significantly after bleomycin treatment, indicating that C/EBP*ζ* expression in lung macrophages has no effect on pulmonary fibrosis [45]. However, a recent report has shown that bleomycin treatment in C/EBP*ζ*^−/−^ mice results in lung ECM deposition associated with an increased number of Arg1-positive macrophages (M2) that activate lung fibroblasts, indicating that C/EBP*ζ* can inhibit M2 polarization to alleviate lung fibrosis [97]. Conversely, another recent study has shown that C/EBP*ζ* deficiency represses M2 macrophage polarization in lung tissues during bleomycin-induced pulmonary fibrosis, thereby attenuating TGF-*β*1 secretion [254]. In particular, C/EBP*ζ* loss promotes SOCS1 and SOCS3 expressions to repress the STAT6/PPAR*γ* signaling, which is essential for M2 macrophage polarization [254]. The varying role of macrophage C/EBP*ζ* in lung fibrosis may be due to differences in processing, indicators of macrophage polarization, and detection methods. Therefore, further studies should investigate the role of macrophage C/EBP*ζ* in pulmonary fibrosis.

In short, although C/EBP*ζ* is mainly located in alveolar epithelial cells with profibrotic effect on lung, C/EBP*ζ* in fibroblasts, LR-MSCs, and macrophages are participated in regulation of lung fibrosis through various signaling pathways.

### 3.3. C/EBPs in Kidney Fibrosis

**Figure 5:**
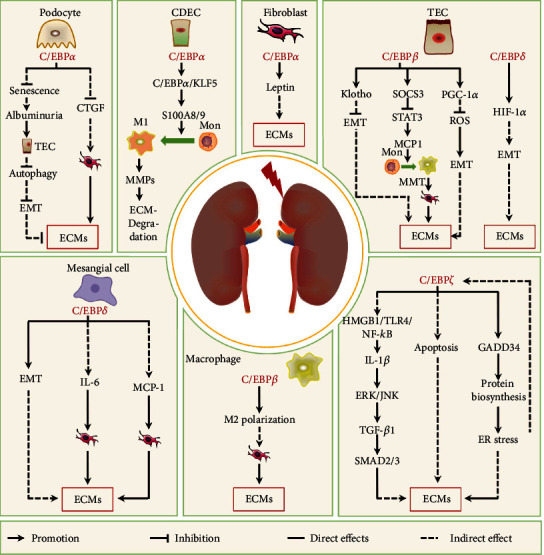
The mechanisms of C/EBPs in kidney fibrosis. C/EBP*α* proteins in podocytes and collecting duct epithelial cells (CDECs) can inhibit renal fibrosis through various mechanisms whereas it in fibroblasts promotes kidney fibrosis indirectly. C/EBP*β* in tubular epithelial cells (TECs) exerts antifibrotic effects while renal macrophage C/EBP*β* possesses profibrotic effects on kidney. C/EBP*δ* proteins in both TECs and mesangial cells exhibit profibrotic effects on kidney. In renal tissues, C/EBP*ζ* upregulation induced by ER stress can promote fibrogenesis.

Kidney fibrosis is the mainly histopathologic manifestation of various chronic kidney diseases (CKDs). Various pathophysiologic characteristics underlying kidney fibrosis are shared with other fibrotic diseases such as cirrhosis and IPF, including injury, inflammation, myofibroblast activation and migration, and ECM deposition and remodeling [255]. In addition, tubular epithelial cells (TECs) and macrophages can promote renal fibrosis through EMT and macrophage-to-myofibroblast transition (MMT), respectively [256, 257]. Mesangial cells, podocytes, and collecting duct epithelial cells (CDECs) are also involved in kidney fibrosis under various stimuli [36, 226, 258]. C/EBP proteins are expressed in these cells and involved in their regulatory effects in renal fibrosis. This section reviews the roles of C/EBPs in these cells to kidney fibrosis (Figure 5).

#### 3.3.1. C/EBP*α*

C/EBP*α* is broadly expressed in the kidneys [259], indicating that it may also play an important role in renal fibrosis. Similar to fibrosis in other organs, fibroblast activation is a central event in renal fibrogenesis [8]. Aristolochic acid, a botanical toxin associated with the development of renal fibrosis, upregulates the DNA binding activity of C/EBP*α* through the AKT/mTOR pathway in kidney fibroblasts [260]. Increased C/EBP*α* activity, not protein expression, directly upregulates the expression of Leptin [260], which is considered a cofactor of TGF-*β* activation that enhances the TGF-*β* signaling in normal rat kidney fibroblasts [261]. Thus, C/EBP*α* expression in fibroblasts indirectly and positively regulates the renal fibrosis.

A recent study shows that C/EBP*α* is mainly expressed in podocytes [259]. Podocyte-specific C/EBP*α* deletion exacerbates renal fibrosis caused by aging [36], indicating that C/EBP*α* in podocytes can inhibit age-induced renal fibrosis. In addition, C/EBP*α* deletion in podocytes aggravates their senescence while C/EBP*α* overexpression inhibits podocyte senescence induced by adriamycin (also known as doxorubicin, an anticancer drug) [36]. In aging mice, podocyte senescence worsens glomerulosclerosis and subsequent albuminuria exacerbates senescent tubular cell EMT by suppressing autophagy, resulting in renal fibrosis [36]. C/EBP*α* overexpression also reduces adriamycin-induced increases CTGF mRNA expression in podocytes [36]. Secreted CTGF can activate surrounding fibroblasts that synthesize and secrete ECM, thereby promoting the occurrence and development of fibrosis [262]. Together, these studies indicate that C/EBP*α* in podocytes inhibits kidney fibrosis through various manners.

C/EBP*α* in CDECs is also involved in tubulointerstitial fibrosis. A previous report has shown that C/EBP*α* regulates the transcription factor Krüppel-like factor 5 (KLF5) during kidney inflammatory responses to injury [263]. KLF5 is mainly expressed in CDECs, and KLF5 haploinsufficiency in CDECs reduces the protein level of C/EBP*α*, thereby inhibiting KLF5/C/EBP*α* complex formation [263]. This complex induces the production of the chemotactic proteins S100A8 and S100A9, which drive monocytes to the kidneys and encourage their polarization into M1-type (CD11b^+^F4/80^lo^) macrophages [263]. M1 macrophages inhibit fibrosis in multiple organs not only by secreting MMPs to directly degrade ECM but also by secreting inflammatory factors to inhibit fibroblast activation and reduce ECM synthesis [264, 265]. Thus, C/EBP*α* expression in CDECs may inhibit renal fibrosis caused by unilateral ureteral obstruction (UUO, a common form of upper urinary tract obstruction can lead to fibrosis) [42].

Together, these studies suggest that C/EBP*α* can positively or negatively regulate the pathological process of renal fibrosis in different cells through various indirect ways. Further studies may be required to elucidate the roles of C/EBP*α* in different cell types of kidneys during physiological and pathological processes, including renal fibrosis.

#### 3.3.2. C/EBP*β*

C/EBP*β* level is decreased in the kidney tissue of animal models of fibrosis, including diabetic nephropathy and UUO model [266–268], indicating a negative correlation between C/EBP*β* levels and renal fibrosis. In TECs stimulated with TGF-*β*1, TNF-*α*, H_2_O_2_, or high glucose, the protein levels or transcriptional activity of C/EBP*β* and its targets genes, including *Pgc1a*, *Klotho*, tubulointerstitial nephritis antigen (*Tinag*), and suppressor of cytokine signaling 3 (*Socs3*) are decreased [87, 266–268]. Decreased PGC-1*α* levels in TECs from mice with UUO cause mitochondrial dysfunction and ROS production, leading to EMT and subsequent ECM production [188, 269]. In addition, EMT and ECM production can be promoted by Klotho downregulation by miR-34a in UUO mice, which eliminates its inhibition of FGF2, TGF-*β*1, and Wnt signal pathways in TECs [270]. High glucose levels decreased SOCS3 in TECs, thereby removing the inhibition of STAT3 activation, increasing monocyte chemoattractant protein-1 (MCP-1) production, and inducing monocyte infiltration into the kidneys [266]. Meanwhile, bone marrow-derived macrophages from injured kidneys can be converted into *α*-SMA^+^ myofibroblasts through the MMT, thus contributing toward pathogenic collagen production during kidney fibrosis [5].

In addition, C/EBP*β* expression is elevated during IL-4-induced M2 polarization of macrophages [271]. C/EBP*β* overexpression has been reported to rescue decreased Arg1, Ym1, and Fizz1 expressions in TSC complex subunit 1- (TSC1-) deficient macrophages treated with IL-4 [189]. These reports indicate that C/EBP*β* regulates macrophage M2 polarization. M2 macrophages can promote renal fibroblast activation by secreting fibrogenic factors such as IL-10 and TGF-*β*1 [271].

In brief, C/EBP*β* upregulation in TECs inhibits renal fibrosis through various mechanisms, while increased C/EBP*β* proteins in kidney macrophages can induce M2 polarization to activate fibroblasts, thus promoting fibrosis.

#### 3.3.3. C/EBP*δ*

Previous studies have shown that C/EBP*δ* levels are increased in kidney tissues from animal models of UUO, diabetic nephropathy, and hypoxic kidney [26, 272, 273], which are closely related to renal fibrosis, indicating that elevated C/EBP*δ* levels may participate in kidney fibrogenesis. In TECs, C/EBP*δ* expression is induced by high glucose, IL-1*β*, or hypoxia via the NF-*κ*B pathway and then directly binds to the *Hif-1a* promoter and enhances its expression [272, 273]. High HIF-1*α* levels promote renal interstitial fibrosis by inducing EMT in TECs [272]. In mesangial cells, C/EBP*δ* levels are upregulated by various factors, including IL-1*β*, LPS, TNF-*α*, and PDGF [274, 275], and can mediate their *trans*-differentiation into myofibroblasts by directly upregulating *α*-SMA expression, thereby promoting the progression of renal fibrosis [276]. In addition, high C/EBP*δ* levels in mesangial cells can induce the expressions of IL-6 and MCP-1 [275], which participate in renal fibrogenesis through multiple mechanisms including activating fibroblasts [277, 278]. Together, these studies indicate that C/EBP*δ* upregulation in TECs and mesangial cells can promote the occurrence and development of renal fibrosis.

#### 3.3.4. C/EBP*ζ*

Under normal physiological conditions, C/EBP*ζ* is mainly expressed in renal tubular epithelial cells [279]; however, C/EBP*ζ* is upregulated in tubular epithelial cells and glomerular endothelial cells under injury stimuli [279–281]. Accumulating evidence has shown that C/EBP*ζ* expression is upregulated in fibrotic kidney tissues from patients with chronic kidney diseases and obesity, and in animal models of kidney injury, C/EBP*ζ* deficiency attenuates renal fibrosis in mouse model UUO, Ang II/deoxycorticosterone acetate/salt, and diabetic nephropathy [279, 282–285]. These studies suggest that C/EBP*ζ* can promote kidney fibrogenesis.

Various *in vivo* and *in vitro* studies have shown that decreased C/EBP*ζ* expression caused by C/EBP*ζ* gene loss, siRNA, or ER stress inhibitors alleviates the apoptosis of renal cells, macrophage infiltration in kidney tissue, and TGF-*β*1 expression [282, 283, 285, 286]. Mechanistically, C/EBP*ζ* upregulation promotes renal fibrosis through apoptosis and C/EBP*ζ* expression induced in renal tissues by UUO can activate the HMGB1/TLR4/NF-*κ*B signaling pathway to stimulate IL-1*β* production, which enhances TGF-*β*1 production via the ERK/JNK pathway and accelerates renal fibrosis by TGF-*β*1/SMAD2/3 signaling [282].

In addition, as the main downstream enforcer of ER stress, C/EBP*ζ* can regulate ER stress. A previous study showed that C/EBP*ζ* upregulation in primary mouse embryo fibroblasts induced by tunicamycin can increase *Gadd34* expression by binding to its promoter, which encodes the regulatory subunit of an eIF2*α*-specific phosphatase complex that promotes global proteins biosynthesis [287]. Accelerated protein biosynthesis results in unfolded and misfolded proteins that cause ER stress under pathophysiological conditions to promote fibrosis. This positive feedback between C/EBP*ζ* and ER stress may occur in various tissues under persistent pathological irritation to promote the progression of injuries or diseases.

These exerting evidence shows that C/EBP*ζ* upregulation can promote renal fibrosis through various mechanisms. However, these studies were carried out in kidney tissue rather than cells. Considering that C/EBP*ζ* is ubiquitously expressed [31] and can be detected in renal parenchymal cells such as podocytes and inflammatory/immune cells such as macrophages [288, 289], further research is required to investigate the roles of C/EBP*ζ* of different renal cell types in kidney fibrosis.

### 3.4. C/EBPs in Heart Fibrosis

**Figure 6:**
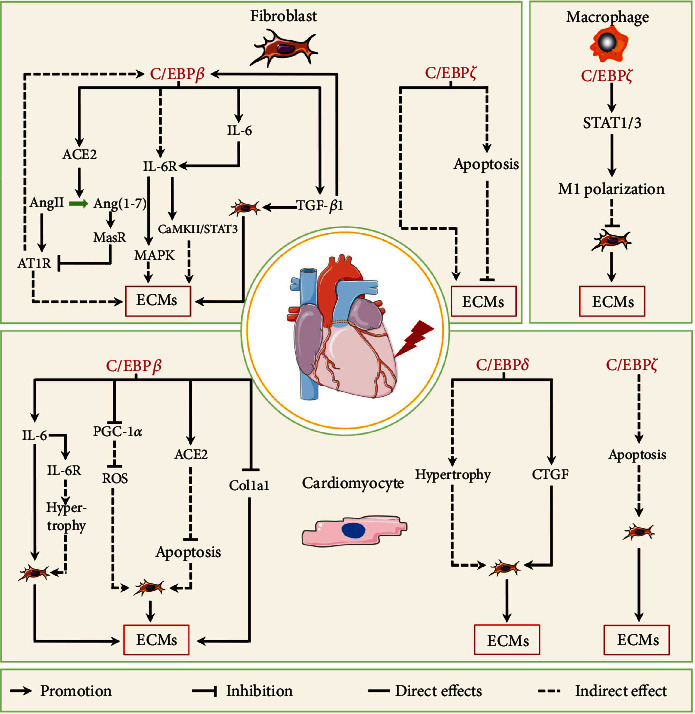
The mechanisms of C/EBPs in heart fibrosis. Among C/EBPs, C/EBP*β*, C/EBP*δ*, and C/EBP*ζ* participate in heart fibrosis. C/EBP*β* in cardiac fibroblasts and cardiomyocytes can promote or inhibit cardiac fibrosis depending on different stimuli. C/EBP*δ* of cardiomyocytes exerts antifibrotic effects on heart fibrosis, while C/EBP*ζ* in various cells possesses different roles in cardiac fibrosis.

Heart fibrosis is a common pathophysiological manifestation of most cardiovascular diseases that are the leading cause of death around the world [112]. Cardiac fibrosis is commonly categorized two types: reactive interstitial fibrosis and replacement fibrosis [2, 112]. Reactive interstitial fibrosis occurs in interstitial and perivascular spaces without significant cardiomyocyte loss and has similar fibrogenic responses to other tissues; replacement fibrosis occurs at the site of cardiomyocyte death and replaces with ECM and noncardiomyocyte to maintain heart integrity [2, 112]. In the heart, resident cardiac fibroblast differentiation into myofibroblasts is a key cellular process that drives the fibrotic response in many different conditions associated with heart failure [290, 291]. Cardiomyocytes are also critical contributors to the myocardial fibrotic response and can exhibit a fibrogenic program that can product ECM or lead to fibroblast activation under various pathophysiologic conditions [291, 292]. In addition, other cell types such as macrophages is implicated in fibrotic remodeling of the heart [183]. Here, we discuss the roles of C/EBPs in these cells during cardiac fibrosis (Figure 6).

#### 3.4.1. C/EBP*β*

C/EBP*β* is detected in normal heart tissue under physiologic conditions; however, it can be downregulated in cardiomyocytes by endurance exercise, resulting in cardiomyocyte hypertrophy and proliferation without fibrosis [86]. Although C/EBP*β* may be involved in the physiologic remolding of cardiomyocytes, the relationship between change in C/EBP*β* protein level or activity and cardiac fibrosis under pathological conditions remains unclear. DCM can induce severe fibrosis and inhibit C/EBP*β* expression in heart tissue [34, 293], while C/EBP*β* overexpression can inhibit cardiac fibrosis caused by DCM [34]. In addition, enhanced cardiac fibrosis, decreased cardiac functions, and high heart C/EBP*β* protein levels have been observed in models of experimental autoimmune myocarditis (EAM), spontaneously hypertensive rats, and transverse aortic constriction (TAC) [38, 86, 294]. Furthermore, C/EBP*β* knockdown attenuates Col1al, Col3a1, and *α*-SMA expressions in the heart tissue of EAM rats [38], suggesting that C/EBP*β* in various cell types may affect cardiac fibrosis in different ways during different disease states.

C/EBP*β* exerts different effects on cardiac fibroblasts under various pathological stimuli. Some studies have reported a negative correlation between C/EBP*β* levels in cardiac fibroblasts and cardiac fibrosis. For instance, C/EBP*β* expression is downregulated in the cardiac fibroblasts with high Col1al, Col3a1, and TGF-*β*1 expression induced by high glucose, whereas C/EBP*β* overexpression in activated cardiac fibroblasts significantly attenuates ECM deposition *in vitro* [34]. Furthermore, C/EBP*β* binds to the angiotensin-converting enzyme 2 (*Ace2*) promoter and activates its expression, which catalyzes angiotensin II (Ang II) cleavage into Ang (1-7) [34, 295]. Ang II then binds to the angiotensin II type I receptor (AT1R) on the surface of fibroblasts, leading to ECM expression and secretion in various ways [296], whereas Ang (1-7) alleviates cardiac fibrosis and heart dysfunction by binding to and activating its receptor (MasR), thereby downregulating AT1R, AT2R, and ACE [34, 297]. However, other reports have shown that C/EBP*β* is involved in the activation of cardiac fibroblasts. As an important fibrogenic factor, TGF-*β*1 can induce C/EBP*β* protein expression of in cardiac fibroblasts; however, lentivirus-mediated C/EBP*β* silencing can inhibit the upregulation of inflammatory factors and cytoskeletal proteins and the differentiation of cardiac fibroblasts caused by TGF-*β*1 [38]. Furthermore, *Tgf-b1* is positively regulated by C/EBP*β* in cardiac fibroblasts [298], forming a positive feedback loop that may play an important role in the activation of cardiac fibroblasts. In addition, norepinephrine can induce C/EBP*β*, IL-6, and IL-6R expressions in nonmyocytes (predominantly of cardiac fibroblasts), with *Cebpb* mRNA levels elevating earlier than *Il-6* and *Il-6r* mRNA levels *in vivo* [299]. C/EBP*β*, also known as nuclear factor for IL-6 expression (NF-IL6), can bind to the *Il-6* promoter and upregulate its expression [300], indicating that C/EBP*β* can upregulate IL-6 expression in cardiac fibroblasts. Autocrine or paracrine IL-6 binds to its receptor IL-6R on the surface of cardiac fibroblasts, leading to ECM expression and deposition via the MAPK and CaMKII-STAT3 pathways [288].

Although C/EBP*β* has been reported to regulate cardiac fibrosis in cardiomyocytes, but its role is paradoxical. Recently, it has reported that C/EBP*β* overexpression can attenuate high glucose-induced cardiomyocytes apoptosis by upregulating ACE2 expression, which alleviates cardiac fibroblast activity and ECM production [34]. Compared to p38*α*-knockout cardiomyocytes, the DNA binding activity of C/EBP*β* to the *Col1α1* promoter is enhanced in wild-type cardiomyocytes, inhibiting *Col1α1* transcription [301]. These studies suggest that C/EBP*β* negatively regulates cardiac fibrosis in cardiomyocytes; however, C/EBP*β* overexpression in primary cardiomyocytes has been reported to inhibit peroxisome proliferator-activated receptor *γ* coactivator-1 alpha (*Pgc1a*) mRNA and protein levels in fibrotic heart tissue from spontaneously hypertensive rats [86, 294], indicating that C/EBP*β* can negatively regulate PGC-1*α* expression. PGC-1*α* is an important coactivator that regulates mitochondrial biogenesis and function in various organs and tissues [302]. PGC-1*α* upregulation in cardiomyocytes reduces TAC-induced heart fibrosis by inhibiting the mitochondrial unfolding protein response [303]. In addition, C/EBP*β* can directly increase IL-6 production in cardiacmyocytes [299]. IL-6 not only binds to IL-6R on the surface of cardiomyocytes and induces myocardial hypertrophy via the MAPK and CaMKII-STAT3 pathways but also binds to IL-6R on the surface of cardiac fibroblasts and activates them [299]. Thus, C/EBP*β* appears to positively regulate cardiac fibrosis in cardiomyocytes.

In brief, the relative contribution of C/EBP*β* various cell types in heart fibrosis may be dependent on the underlying cause of cardiac injury and different expression of its isoforms.

#### 3.4.2. C/EBP*δ*

Mice lacking C/EBP*δ* are viable and healthy and exhibit no abnormalities, indicating that C/EBP*δ* is not vital for survival [53, 97]. In most cells including cardiomyocytes, C/EBP*δ* expression is low under normal physiological conditions but can be rapidly induced by external stimuli [31]. Since C/EBP*δ* plays physiological roles in cell differentiation, proliferation, apoptosis, energy metabolism, and inflammation by regulating the expression of specific genes [31, 42], it may affect the pathogenesis of diseases such as cardiac fibrosis.

C/EBP*δ* expression is upregulated in the heart by adverse stimuli such as hypertension, LPS, and norepinephrine, similar to the liver [299, 304, 305]. Studies of cardiac fibrosis have mainly focus on the role of C/EBP*δ* in cardiomyocytes. For instance, enhanced C/EBP*δ* transcriptional activity in cardiomyocytes from Rad knockout mice under TAC has been shown to activate CTGF expression, which stimulates cardiac fibroblasts to produce more ECM [306]. In addition, C/EBP*δ* protein levels can be induced by IL-6 and mediated by STAT3 in cardiomyocytes, leading to cardiac hypertrophy which can cause cardiac fibrosis through multiple mechanisms [299, 307]. These results support the conclusion that C/EBP*δ* positively regulates the occurrence and development of cardiac fibrosis.

#### 3.4.3. C/EBP*ζ*

An extensive body of literature has shown that C/EBP*ζ* is involved in cardiomyocyte apoptosis, cardiac hypertrophy, and heart fibrosis. Various stimuli such as TAC, myocardial infraction (MI), and diabetes activate ER stress by increasing C/EBP*ζ* expression in myocardial fibrosis [308–310]. In addition, C/EBP*ζ* knockout mice display decreased cardiomyocyte apoptosis and subdued cardiac fibrosis under TAC, ischemia-reperfusion (I/R) injury, and Ang II-induced hypertension compared to wild-type mice [311–313]. Thus, C/EBP*ζ* may positively regulate cardiac fibrogenesis. Mechanistically, the upregulation of C/EBP*ζ* by multiple factors, including phenylephrine, doxorubicin, and high glucose, has been reported to induce cardiomyocyte apoptosis [308, 314, 315], which can lead to organ remodeling and fibrosis after insult by activating fibroblasts [21].

The role of C/EBP*ζ* in noncardiomyocytes in fibrosis remains somewhat contradictory. Consistent with its role in cardiomyocytes, C/EBP*ζ* upregulation by I/R or tunicamycin can cause apoptosis of cardiac fibroblasts, thereby mitigating fibrosis [316]. However, increased C/EBP*ζ* levels in primary cardiac fibroblasts explored to Ang II can increase the expression of ECM proteins, as confirmed by a decrease in C/EBP*ζ* expression and fibrotic markers after treatment with an ER stress inhibitor [317]. In cardiac macrophages, C/EBP*ζ* upregulation by hypoxia-induced mitogenic factor under MI or C/EBP*ζ* overexpression can increase STAT1 and STAT3 phosphorylation, which can promote macrophage M1 polarization and increase production of proinflammatory cytokines that reduce the viability and activation of cardiac fibroblast [318]. These studies indicate that C/EBP*ζ* expression in noncardiomyocyte cells may contribute to cardiac fibrosis in different ways under diverse stimuli.

### 3.5. C/EBPs in Neural Fibrosis

**Figure 7:**
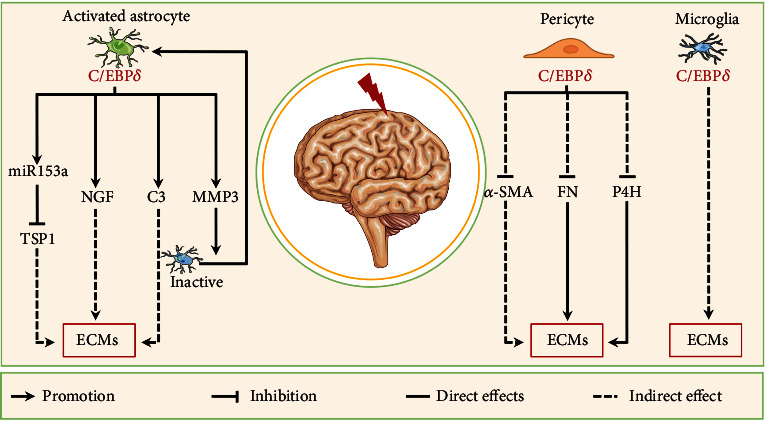
The mechanisms of C/EBPs in neural fibrosis. Among C/EBPs, C/EBP*δ* is involved in neural fibrosis. C/EBP*δ* in microglia can promote neural fibrosis whereas it in pericytes inhibits fibrosis of the nervous system. C/EBP*δ* of activated astrocytes exerts the profibrotic or antifibrotic effects on neural fibrosis depending on various stimuli.

Central nervous system (CNS) trauma generates cellular debris, activates resident cells, infiltrates circulating immune cells, and eventually forms two distinct scars: glial scar and fibrotic scar [319, 320]. As a unique form in CNS, glial scar is mainly formed by the accumulation of reactive astrocytes in injured sites [320]. Reactive astrocytes, characterized by the increased expression of glial fibrillary acidic protein, surround the lesion and separate the injured area from normal tissue [320, 321]. Fibrotic scar, located in the injured core, is characterized by the presence of fibroblasts and ECM deposition [320]. Although the similar of CNS fibrotic scar to other organ fibrosis, its formation process may be quite different due to the unique CNS environment [320]. Various cell types in CNS such as fibroblasts, astrocytes, microglia, pericytes, and endothelial cells play important roles in formation of fibrotic scar [320]. C/EBP*δ* is detected in astrocytes, microglia, and pericytes and has a crucial role in CNS function [31]. ECM deposition and C/EBP*δ* levels are observed in the spinal cord of patients with amyotrophic lateral sclerosis (ALS) and the brains of patients with Alzheimer's disease, indicating that C/EBP*δ* is involved in neurological fibrosis [322, 323]. Here, we discuss the roles of C/EBP*δ* in astrocyte, microglia, and brain pericyte in CNS fibrosis (Figure 7).

Astrocytes are a highly abundant cell type in the CNS, with astrocytes to neurons ratio of 1 : 3 in the cortex of mice and rats and 1.4 : 1 in the human cortex [324]. Astrocytes maintain CNS homeostasis under physiological conditions and are activated by CNS injury and diseases [325]. Reactive astrocytes (activated) are a major source of chondroitin sulfate proteoglycans, a family of ECM proteoglycans, and can therefore contribute toward scar tissue by increasing ECM protein deposition [326, 327]. C/EBP*δ*-deficient mice display reduced glial scar formation after moderate spinal cord contusion injury at the mid-thoracic level, indicating that C/EBP*δ* promotes glial scar formation [328]. Furthermore, C/EBP*δ* expression is increased in astrocytes stimulated with factors such as IL-1*β*, IL-6, TNF-*α*, or prostaglandin E2 [322, 328, 329]. High C/EBP*δ* levels can directly upregulate the expression of MMP3, which promotes the migration of inactive astrocytes to the injured area, resulting in glial scar formation [328]. However, C/EBP*δ* can also bind to the *miR-153a* promoter and upregulate its expression to repress the transcription of thrombospondin 1 (TSP1) via its 3′UTR [191, 329]. In addition, C/EBP*δ* directly upregulate Complement 3 (C3) expression in astrocytes [330]. Besides, C/EBP*δ* knockout mice display a complete loss of nerve growth factor (NGF) induction in the cerebral cortex under *β*2-adrenergic receptor agonist treatment, with *in vitro* experiments confirming that *NGF* is a direct downstream target gene of C/EBP*δ* [331]. Although TSP1, C3, and NGF play an important role in fibrotic diseases, such as renal fibrosis and cardiac fibrosis [332–334], further research is required to determine whether these factors mediate the regulation of astrocyte C/EBP*δ* in CNS fibrosis.

Pericytes are distributed throughout the body but have a higher density in the CNS [335]. Pericytes expressing the fibroblast markers *α*-SMA, fibronectin, and prolyl-4-hydroxylase (P4H) can give rise to fibroblast-like cells (type A pericytes) that constitute the fibrotic compartment of scars and are required for fibrosis and ECM deposition [336, 337]. C/EBP*δ* can be detected in brain pericytes and induced by IL-1*β* in a concentration- and time-dependent manner [258, 338]. In addition, pericytes with high C/EBP*δ* levels possess lower *α*-SMA, fibronectin, and P4H expression, indicating that C/EBP*δ* can negative regulate the differentiation of pericytes into fibroblasts.

Macrophages in the brain, also known as microglia, are critical for orchestrating the injury response in the CNS [339]. Nuclear C/EBP*δ* levels are increased in microglia from ALS patients and G93A-SOD1 mice (animal model of ALS), indicating that microglial C/EBP*δ* may promote fibrosis in CNS [323, 340]. However, the detailed mechanisms through which C/EBP*δ* regulates fibrosis in microglia require further study.

Available reports have shown that C/EBP*δ* upregulation exerts profibrotic or antifibrotic effect in CNS fibrosis depending on different cell types, but the clear and definite mechanisms are unclear. Next studies could be required to uncover the roles of C/EBP*δ* in neural fibrosis through *Cebpd* gene-modified animal models in specific CNS cells.

### 3.6. C/EBPs in Fibrosis of Other Tissues

#### 3.6.1. Muscle Fibrosis

**Figure 8:**
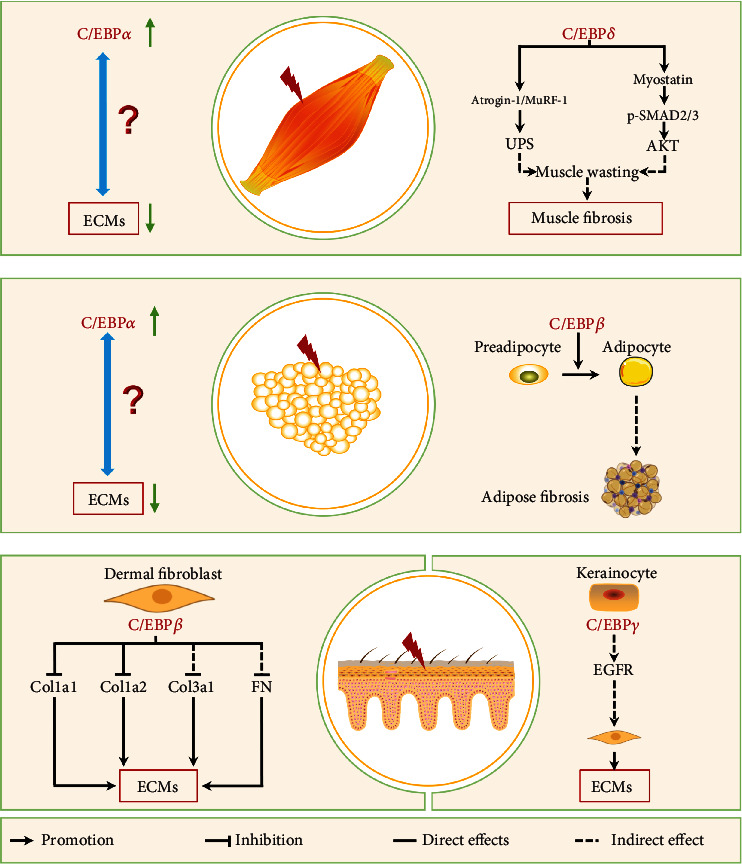
The relationships and mechanisms of C/EBPs in fibrosis of other tissues. The roles of C/EBPs in fibrosis of skeletal muscle, fat, and skin are shown. Under the stimuli, C/EBP*α* is negatively correlated with skeletal muscle fibrosis and fat fibrosis. Upregulated C/EBP*β* can promote the differentiation of adipocytes leading to fibrosis and inhibit the ECM production in dermal fibroblasts to suppress skin fibrosis. C/EBP*γ* and C/EBP*δ* can promote skin fibrosis and skeletal muscle fibrosis through various mechanisms, respectively.

As an essential component of skeletal muscle, ECM provides a framework structure that holds myofibers, blood capillaries, and nerves to support the force transmission, maintenance, and repair of muscle fibers [341]. Skeletal muscle fibrosis often occurs after major muscle trauma or extensive surgical reconstructions and also is a hallmark of muscular dystrophies and aging [341, 342]. Fibrosis of skeletal muscle can impair muscle function, inhibit muscle regeneration after injury, and increase muscle susceptibility to reinjury [341, 343]. The predominant cell type responsible for ECM deposition in muscle fibrosis is fibroblast [343]. In addition, several signaling pathways have been reported to play an important roles in promoting muscle fibrosis, including TGF-*β*1, CTGF, Myostatin, Wnt, PDGF, and vascular endothelial growth factor (VEGF) [341, 343]. C/EBP*α* and C/EBP*δ* can be detected in muscle tissue and play an important role in fibrosis of skeletal muscle [282, 286]. Here, we discuss the relationship and roles of C/EBP*α* and C/EBP*δ* in skeletal muscle fibrosis (Figure 8).

C/EBP*α* can be detected in skeletal muscle tissue, indicating that it may affect muscle fibrosis [344, 345]. In a full-thickness supraspinatus tear rat model, simvastatin was reported to reduce *Cebpa* gene expression, decrease the mRNA levels of ECM synthesis-, fibrosis-, and fibroblast proliferation-related genes, and inhibit collagen accumulation by 50% in muscles [346], indicating that C/EBP*α* plays a significant role in muscle fibrosis. In addition, unloading conditions were found to decrease the fibroprogenitor markers' expression and C/EBP*α* mRNA levels in a glycerol model of muscle regeneration [347], further suggesting that C/EBP*α* expression correlates negatively with muscle fibrosis. In addition, C/EBP*δ* is detected in muscle tissue and induced by activated STAT3 during catabolic conditions, such as chronic kidney disease and cancer cachexia [348, 349]. C/EBP*δ* directly upregulates Myostatin expression by binding to its promoter, which activates the SMAD2/3/AKT pathway and causes muscle wasting [348, 349]. In addition, elevated C/EBP*δ* increases Atrogin-1 and MuRF-1 levels, triggering the ubiquitin-proteasome system to accelerate muscle wasting and fibrotic deposition [349, 350]. These studies indicate that C/EBP*α* has an antifibrotic effect in skeletal muscle fibrosis, while C/EBP*δ* promotes the fibrosis of skeletal muscle under different stimuli.

#### 3.6.2. Adipose Tissue Fibrosis

Adipose tissue is a complex heterogeneous tissue composed of adipocytes and nonadipocytes. Excess lipids and adipocyte hypertrophy can lead to hypoxia and inflammation in fat tissue during obesity [351]. Adipose tissue hypertrophy causes hypoxia to induce HIF-1*α* expression, upregulating many extracellular factors such as collagens that establish and remodel ECM. [352]. In cellular level, fibroblasts and inflammatory cells such as lymphocytes, mast cells, and macrophages play vital roles in producing depot-specific ECM and adipose tissue fibrosis [351]. C/EBP*α* and C/EBP*β* play an important role in maintaining adipose tissue homeostasis and also participate in fibrosis of adipose tissue (Figure 8).

Collagen accumulation is increased in the adipose tissue of patients with HIV-1-related lipoatrophy, whereas C/EBP*α* expression is reduced [353]. In addition, cancer cachexia can lead to the loss of adipose tissue and increased fibrosis in the tissue matrix, accompanied by a significant decrease in C/EBP*α* mRNA and protein expression [354]. Besides, high-dose G-CSF also causes severe fibrosis and downregulates C/EBP*α* expression in fat grafts [355]. These studies indicate that C/EBP*α* negatively correlates with adipose fibrosis caused by different diseases or stimuli. Besides, as a regulator of the adipogenic/lipogenic transcription, C/EBP*β* overexpression promotes the differentiation of preadipocytes into adipocytes, causing adipocyte hypertrophy [356]. Adipose tissue hypoxia is induced by adipocyte hypertrophy and hyperplasia, resulting in inflammation and fibrosis [357], indicating that C/EBP*β* positively correlates with adipose fibrosis. Further studies may be required to explore the detailed role of C/EBP*α* and C/EBP*β* in adipocytes and nonadipocytes during adipose tissue fibrosis and whether other C/EBPs affect the fibrosis process of adipose tissue.

#### 3.6.3. Skin Fibrosis

Like other organ fibrosis, skin fibrosis is the excessive ECM deposition in the dermis and occurs following tissue injury such as burns, trauma, infection, surgery, and radiation, leading to scars that limit movement and cause significant psychological distress for patients [13, 358]. Many molecular pathways have been implicated in the development of skin fibrosis, including TGF-*β*, Wnt, and epidermal growth factor receptor (EGFR) signaling pathways [359]. Furthermore, myofibroblasts derived from dermal fibroblasts, pericytes, dermal adipocytes, and perivascular cells play a vital role in skin fibrosis [13, 360]. In addition, multiple cell types in skin such as keratinocytes, T cells, and macrophages have been implicated in skin fibrosis [13]. Recent studies have shown that C/EBP*β* and C/EBP*γ* participated in skin fibrosis through different mechanisms [249, 250] (Figure 8).

In dermal fibroblasts, *Col1α1* and *Col1α2* are target genes of C/EBP*β* and increased C/EBP*β* expression induced by inflammatory factors, such as interferon beta (IFN-*β*) and IFN-*γ*, inhibits not only Col1*α*1 and Col1*α*2 expression but also the levels of Col3*α*1 and fibronectin, which suppresses ECM deposition [210, 211, 361]. In addition, a recent report showed that C/EBP*γ* can promote skin wound healing, indicating that C/EBP*γ* may play an important role in fibroblast activation. Wounding induces C/EBP*γ* expression in keratinocytes, while inhibiting C/EBP*γ* using siRNA can impair wound healing *in vivo* and *in vitro* [362]. C/EBP*γ* silencing also inhibits the migration of keratinocytes induced by EGF or serum, whereas C/EBP*γ* overexpression enhances their migration to EGF or serum via regulating the phosphorylation of EGFR, which affects cell migration and epidermal wound healing [362]. There is strong evidence that keratinocytes activate fibroblasts and cause them to produce growth factors, which in turn increases keratinocyte proliferation [363]. Activated fibroblasts are critical in creating ECM structures that support the other cells involved in effective wound healing [364].

These studies only detect C/EBP levels of mRNA and protein expression at the tissue level, not the cell level, raising several questions: (1) Is the decrease in C/EBPs and increase of tissues fibrosis a concomitant or causal relationship? (2) Does the transcriptional activity of C/EBPs change when C/EBPs protein levels decrease? (3) What are the roles of C/EBPs in the various cell types found in muscle, fat, and skin during fibrosis? More in-depth research is needed in the future to answer these questions.

In summary, C/EBPs can exert different effects on fibrosis in the same organ or tissue fibrosis. Some C/EBPs inhibit fibrosis in various organs; for instance, C/EBP*α* expression is decreased in several types of organ fibrosis, except in renal fibrosis, and C/EBP*α* overexpression lessens fibrosis, indicating the antifibrotic effect of C/EBP*α* under most conditions. Conversely, some C/EBPs accelerate fibrosis in various organs. For example, C/EBP*δ* expression and activity are increased in different types of nonliver fibrosis, with C/EBP*δ* deficiency inhibiting fibrosis, suggesting C/EBP*δ* has profibrotic effects under most conditions. Other C/EBPs, such as C/EBP*β*, can have positive or negative effects on fibrosis depending on the cell type and stimulus. The overall effects of C/EBPs on fibrosis in different organs are summarized in Table 1.

## 4. Crosstalk between C/EBPs and Classical Fibrotic Factors

Studies in recent decades have shown that some conserved fibrotic molecules or classical fibrotic factors drive fibrogenesis in different organs and species. These classical fibrotic factors involve TGF-*β*1, CTGF, and PDGF [1, 2, 14]. The crosstalk between C/EBPs and classical fibrotic factors may play the important role in C/EBPs' regulation of fibrosis. Existing reports focus on the role of crosstalk between C/EBPs and TGF-*β*1 or CTGF in fibrosis, in which we discuss these relations below.

### 4.1. C/EBPs Crosstalk with TGF-*β*1

TGF-*β* has been well-documented as a profibrotic cytokine since its first reported role in stimulating the expression of ECM in fibroblasts [365]. Three separate TGF-*β* isoforms (TGF-*β*1, TGF-*β*2, and TGF-*β*3) have been identified in mammals. These TGF-*β* isoforms share a similar biologically active region and can bind to TGF-*β* receptor 2 (TGFR2), which recruits and activates TGFR1 to activate receptor signaling [15]. In human, TGF-*β*1 was found to be the most abundant isoform and is widely expressed by most cells [366]. TGF-*β* signal acts on various cell types to drive fibrosis through both the SMAD- and non-SMAD-mediated pathways. In addition to tissue fibrosis, TGF-*β*1 also regulates many biological responses, such as cell proliferation, differentiation, autophagy, and immune response [15].

C/EBPs exhibit diverse regulatory relationships with TGF-*β*1 during fibrosis in different organs and can both up- and downregulate TGF-*β*1 activity or expression in different cell types. For example, C/EBP*α* can upregulate Leptin expression, which enhances the TGF-*β*1 signaling in normal rat kidney fibroblasts [260, 261]. Meanwhile, C/EBP*β* directly increases TGF-*β*1 expression in cardiac fibroblasts and pulmonary macrophages and can bind to the *Tfg-b1* promoter and suppress its expression in HSCs [42, 103, 298]. C/EBP*ζ* also upregulates TGF-*β*1 expression through the HMGB1/TLR4/NF-*κ*B/IL-1*β* pathway in renal tissue [282].

TGF-*β*1 can also indirectly modulate the expression or activity of C/EBPs. For example, TGF-*β*1 not only upregulates C/EBP*β* expression in lung fibroblasts and HSCs but also enhances C/EBP*β* activity by promoting its acetylation in alveolar epithelial cells [149, 152, 203, 213]. In tubular epithelial cells, TGF-*β*1 suppresses C/EBP*β* expression through the PDE/cAMP/Epac pathway to regulate mitochondria biogenesis [267], yet in cardiac fibroblasts, TGF-*β*1 treatment inhibits C/EBP*ζ* expression [316]. Furthermore, the positive feedback loop formed by TGF-*β*1 and C/EBP*β* in cardiac fibroblasts and by TGF-*β*1 and C/EBP*δ* in pancreatic stellate cells may accelerate their activation [298, 367]. These research indicates that the crosstalk between C/EBPs and TGF-*β*1 plays an important role in regulation of fibrosis by C/EBPs. The regulatory mechanisms involving C/EBP and TGF-*β*1 are summarized in Table 2.

### 4.2. C/EBPs Crosstalk with CTGF

CTGF (also known as cellular communication network factor 2 (CCN2)) is one of the best-studied members of the CCN family, which is involved in regulating a variety of important biological functions and pathological processes including tissue fibrosis [17]. CTGF was firstly discovered in fibroblasts and endothelial cells and has since been detected in many organs and tissues [368]. In addition to participating in many biological functions, including cell proliferation, differentiation, and adhesion, CTGF drives the onset and progression of fibrosis in many organs and tissues through various mechanisms [17, 368]. CTGF has been consistently associated with fibrotic remodeling in various organs and has been widely used as the marker to detect fibrosis.

As a downstream modulator of TGF-*β*1, CTGF has been implicated in the occurrence and development of fibrosis [17, 369]. Multiple recent studies have examined direct and indirect mechanisms of regulation between C/EBPs and CTGF. For instance, C/EBP*β* has been shown to indirectly upregulate CTGF expression in lung fibroblasts and directly enhance its expression in human alveolar epithelial cells [204, 212]. Moreover, C/EBP*δ* directly increases CTGF expression in cardiomyocytes [306]. However, C/EBP*α* can indirectly suppress CTGF expression in lung fibroblasts and renal podocytes [36, 43]. These studies suggest that CTGF is involved in the regulation of fibrosis by C/EBPs. The regulatory mechanisms involving C/EBPs and CTGF are summarized in Table 3.

## 5. Therapy Strategies Targeting C/EBPs in Fibrosis

Given that C/EBPs play important roles in the pathogenesis of fibrosis, regulating their expression or activity is an attractive strategy for treating fibrotic diseases. Various *in vivo* experiments have shown that C/EBP*α* overexpression can inhibit CCl_4_-induced liver fibrosis [37], C/EBP*β* deficiency in hematopoietic cells can mitigate bleomycin-induced pulmonary fibrosis [190], C/EBP*δ* lost can aggravate UUO-induced renal fibrosis [26], and C/EBP*ζ* deficiency can alleviate TAC-induced cardiac fibrosis [311], thus providing an experimental basis for the realization of this strategy. Here, we have reviewed interventions involving C/EBPs that improve fibrotic diseases, which have been divided into four categories: oligodeoxynucleotides, oligopeptides, clinical medicines, and compounds (Table 4).

Oligodeoxynucleotides and oligopeptides specifically target C/EBPs at the RNA level and protein level. Recent reports have shown that short duplex RNA oligonucleotides can target the promoter regions of genes and mediate their transcriptional activation [126, 370, 371]. Short activating RNA (saRNA) are important RNA oligonucleotides that enhance gene expression through transcriptional and epigenetic alterations [372]. For example, C/EBP*α*-saRNA can increase C/EBP*α* RNA and protein levels in hepatocytes [126]. Since C/EBP*α* expression is mostly reduced in fibrotic diseases, the specific upregulation of C/EBP*α* may improve fibrotic diseases such as hepatic fibrosis. In addition, oligopeptides can bind to target proteins and inhibit their function. For instance, the dominant-negative C/EBP (A-C/EBP) protein expressed by an exogenously specific nucleotide sequence, which possesses a leucine zipper but lacks functional DNA-binding and transactivation domains and forms stable inactive heterodimers with C/EBP*β* to inhibit its transcriptional activation in preadipocytes and adult epicardium, reduce injury-induced cardiac fibrosis, and improve heart function [106, 373]. However, *in vivo* studies of the precise mechanisms and specific delivery systems are required before these advances can be applied to under clinical conditions.

Marketed clinical medicines, such as lipid-lowering drugs, hypoglycemic agents, and antiviral drugs have been shown to improve fibrosis while modulating the expression or activation of C/EBPs at the animal or cellular levels [107, 148, 346]. Clinical trials are needed to verify their efficacy as clinical antifibrosis treatments, as well as in-depth studies of the precise antifibrotic mechanisms, including C/EBP regulation. In addition, various traditional Chinese medicine compounds and monomers, such as echinomycin, tauroursodeoxycholic acid, curcumin, and quercetin have been shown to alleviate fibrotic diseases by regulating C/EBP expression *in vivo* and *in vitro* [169, 286, 356, 374]. Given the complex structures and existing mechanisms reported for these substances, they are likely to regulate C/EBP expression indirectly; however, their antifibrotic effects and the precise mechanisms, including whether they modulate C/EBPs, require further study.

To date, only two drugs have been approved for antifibrotic therapy of IPF: nintedanib and pirfenidone [3]. Nintedanib is an intracellular inhibitor of tyrosine kinases that have been implicated in the pathogenesis of fibrosis [375]. Pirfenidone, initially developed as an anti-inflammatory substance due to its ability to reduce the accumulation of inflammatory cells and cytokines, has been chiefly characterized as an antifibrotic agent that attenuates fibroblast proliferation and differentiation into myofibroblast, as well as the synthesis and deposition of ECM proteins by inhibiting TGF-*β* and other fibrogenic growth factors [376]. Despite recent studies that have elucidated key mechanisms, the precise molecular activities of nintedanib and pirfenidone remain unclear [375, 376], and further research is required to determine whether C/EBPs are involved in the antifibrotic effects of these two drugs.

## 6. Conclusions and Perspectives

In this review, we have mainly summarized a broad range of recent advances on C/EBPs research in the context of fibrosis. These studies have partly revealed the crucial and complex roles of C/EBPs in fibrotic onset and progression in multiple organs. In summary, C/EBP*α* exerts a notable antifibrotic effect in the liver, lung, and kidney fibrosis diseases, while paradoxically promoting fibrosis in the liver of older patients. C/EBP*β* possesses an antifibrotic effect in skin fibrosis, while having a positive correlation with fat fibrosis. C/EBP*γ* can inhibit the lung fibrosis while promoting the skin fibrosis. C/EBP*δ* possesses a profibrotic effect in the heart, lung, kidney, and muscle fibrosis diseases. C/EBP*ζ* exerts a profibrotic effect in kidney fibrosis. Modulating C/EBP expression and/or activity can exert antifibrotic effects in multiple organs; therefore, novel C/EBPs-based therapeutic methods for treating fibrosis have attracted considerable attention.

Despite the encouraging progress in exploring the relationship between C/EBPs and fibrosis, many critical questions still remain unanswered, and more knowledge is needed before C/EBPs are utilized clinically for fibrosis treatment. Most available studies were carried out in animal models of fibrosis, rather than in clinical specimens from fibrosis-related diseases. Definitive clinical evidence on the relationship between C/EBPs and fibrosis is necessary in research targeting C/EBPs for treatment of fibrotic diseases. In the future, more work is needed to determine the changes of C/EBPs (including genetic polymorphisms, mRNAs, proteins, isoforms, and PTM of C/EBPs) in clinical fibrotic specimens from different organs or same organ in different fibrotic states to confirm the role of C/EBPs in fibrosis and the correlation between their changes and fibrotic degree.

Second, the mechanism of C/EBPs in regulating fibrosis requires more in-depth studies. (1) Although fibrosis was previously thought to be irreversible, there is now a growing body of evidence suggesting that fibrosis is reversible in fibrotic diseases under some circumstances. Regulating ECM degradation is an important mechanism under fibrogenesis [12], which can be targeted in novel therapeutic strategies. The most important enzymes which contribute to ECM degradation are MMPs [377]. Some studies have shown that C/EBPs can regulate the expression of MMPs. For instance, C/EBP*α* can induces the expression and secretion of MMP8/9 in neutrophils to mediate ECM degradation of the liver [24]. Considering that MMPs are produced by various cell types and an important role of ECM degradation in fibrosis [12, 377], additional research is needed to explore the roles of C/EBPs in regulating ECM degradation including MMPs during fibrosis. (2) Metabolic dysregulation is increasingly recognized as an important pathogenic process that underlies fibrosis in many organs [3]. Indeed, C/EBPs also paly vital roles in fibrosis caused by metabolic abnormalities. For instance, decreased C/EBP*α* activity inhibits high-fat diet-induced liver fibrosis [75], while overexpression of C/EBP*β* suppresses diabetes-induced cardiac fibrosis [34]. However, increased C/EBP*δ* exacerbates muscle fibrosis in diabetes conditions [348]. These studies indicate that C/EBPs may be involved in metabolic dysregulation. Future investigations of the regulatory relationships between C/EBPs and metabolic homeostasis may expand our understanding of C/EBPs functions and provide further support for fibrosis therapy by targeting C/EBPs. (3) In addition to forming heterodimers between C/EBPs to regulate other's transcriptional functions, they can also bind to the gene promoters to regulate the protein expressions of different members. For instance, the complex of C/EBP*α* and C/EBP*β* binds the Cebpa promoter and active the expression of C/EBP*α* in the liver [73]. Besides, C/EBP members can be detected in the same cell type under fibrosis, such as C/EBP*α*, C/EBP*β*, C/EBP*γ*, C/EBP*δ*, and C/EBP*ζ* in alveolar epithelial cells [196, 212, 223, 225, 242]. These studies indicate that C/EBPs appear to play a coordinated and likely, partially redundant role in many cell types. However, the effects of these coordinated interactions and complementary roles among C/EBPs during fibrotic process remain largely unknown. Additional studies are needed to explore the roles of C/EBP coordination and complementation in various fibrosis-related cell types to clarify the unique and common roles of each member in fibrosis. (4) Growing evidence shows that the nontranscriptional functions of transcription factors also play the important roles in various physiological and pathological processes [378–380]. As an important transcription factor family, some C/EBP isoforms can also interact with other proteins and perform non-transcriptional functions. For example, C/EBP*δ* can bind to FANCD2 and facilitate its nuclear import [66]. More investigations on this topic will not only deepen the understanding of C/EBPs' functions but also clarify the significance of both the transcriptional and nontranscriptional functions of C/EBPs in fibrosis to provide support for subsequent development of antifibrotic drugs targeting these different functions.

Third, considering that C/EBPs are multifunctional transcriptional factors that play different roles among different cell types, more in-depth studies are required to explore how to properly modulate C/EBP expression or activity in certain cell types and at proper stages to maximize the beneficial effects of C/EBPs on fibrosis and avoid unnecessary adverse effects. Technological advances have provided evidence on possible approaches for controlling fibrotic diseases by targeting C/EBP proteins. Coronavirus disease 2019 (COVID-19) heightened interest in the use of mRNA as vaccine and drug [381, 382]. Similar to C/EBP*α*-saRNA or decoy double-stranded oligodeoxynucleotides of C/EBP*β* (C/EBP*β*-dODN), the mRNA of C/EBP family members, when specifically delivered to the tissue, upregulates the expression of specific members or inhibits their transcriptional activity to improve fibrotic disease outcomes [103, 126]. Further research is needed to design and screen mRNA fragments of the C/EBP family members and explore the methods, optimal dosing, timing of administration, and side effects of these therapies. Additionally, advances in drug delivery systems including modified peptide-, albumin-, nanoparticle-, aptamer-, hydrogel-, or antibody-based systems show promise for developing clinical fibrosis management strategies which target C/EBPs.

Fibrosis is a common outcome following organ injury and leads to organ malfunction and potentially death. The existing evidence summarized in this review strengthens the hypothesis that C/EBPs may be effective targets for fibrosis treatment and will serve as a reference for further research in this field.

## Figures and Tables

**Table 1:** The overall effects of C/EBPs in different organ fibrosis.

	Hepatic fibrosis	Pulmonary fibrosis	Renal fibrosis	Cardiac fibrosis	Fibrosis of CNS	Muscle fibrosis	Adipose fibrosis	Skin fibrosis
C/EBP*α*	-	-	**+/-**			-	-	
C/EBP*β*	**+/-**	**+**	-	**+/-**			**+**	-
C/EBP*γ*		-						**+**
C/EBP*δ*	-	**+**	**+**	**+**	**+**	**+**		
C/EBP*ζ*	**+/-**	**+/-**	**+**	**+**				

“+” means positive effect; “-” means negative effect; “**+/-**” means that the effects are not consistent.

**Table 2:** The regulations between C/EBPs and TGF-*β*1.

Organ	Effect	References
*C/EBPs positively regulate TGF-β1 activity or expression*		
Kidney	C/EBP*α* directly upregulated the leptin expression, which enhances the TGF-*β* signaling in normal rat kidney fibroblasts	[260, 261]
Heart	C/EBP*β* can upregulate target gene Tgf-b1 in the cardiac fibroblasts	[298]
Kidney	Overexpression C/EBP*β* polarizes macrophages to M2, which has increased levels of TGF-*β*1	[189, 264]
Pancreas	C/EBP*δ* upregulates TGF-*β*1 expression in pancreatic stellate cells	[367]
Lung	C/EBP*ζ* deficiency repressed the M2 polarization, which then attenuated TGF-*β*1 secretion in macrophages	[254]
Kidney	C/EBP*ζ* upregulates the expression of TGF-*β*1 through the HMGB1/TLR4/NF-*κ*B/IL-1*β* pathway in renal tissue	[282]
*C/EBPs negatively regulate TGF-β1 activity or expression*		
Liver	C/EBP*β* binds to the promoter of TGF-*β*1 and suppresses its expression in HSCs	[103]
*TGF-β1 positively regulate C/EBP expression or activity*		
Liver	TGF-*β*1 increases the expression or activity of C/EBP*β* to promote the expression of collagens in HSCs	[149, 152]
Lung	TGF-*β*1 can upregulate the protein level of C/EBP*β* in the lung fibroblasts	[203]
Lung	TGF-*β*1 enhances the activity of C/EBP*β* through increasing its acetylation to induce the EMT of alveolar epithelial cells	[213]
Pancreas	TGF-*β*1 increases the expression of C/EBP*δ* in pancreatic stellate cells	[367]
*TGF-β1 negatively or not regulate C/EBP expression or activity*		
Lung	TGF-*β*1 treatment did not affect the expression of C/EBP*α* in lung fibroblast	[43]
Lung	TGF-*β*1 inhibits the expression of C/EBP*β* in alveolar epithelial cells	[383]
Kidney	TGF-*β*1 suppresses the expression of C/EBP*β* through the PDE/cAMP/Epac pathway to regulate mitochondria biogenesis in tubular epithelial cells	[267]
Heart	TGF-*β*1 inhibits the expression of C/EBP*ζ* to suppress the apoptosis of cardiac fibroblasts	[316]

**Table 3:** The regulations between C/EBPs and CTGF.

Organ	Effect	References
*C/EBPs positively regulate CTGF activity or expression*		
Lung	C/EBP*β* upregulates the expression of CTGF in lung fibroblasts through ADAM17	[204]
Lung	C/EBP*β* enhances CTGF expression in human alveolar epithelial cells	[212]
Heart	C/EBP*δ* can increase CTGF expression in cardiomyocytes	[306]
*C/EBPs negatively regulate CTGF activity or expression*		
Lung	Overexpression of C/EBP*α* inhibited the mRNA level of CTGF in lung fibroblasts vice versa	[43]
Kidney	Overexpression C/EBP*α* reduced the increased mRNA level of CTGF in podocytes induced by adriamycin	[36]

**Table 4:** The compounds to regulate the expression of C/EBPs in fibrotic diseases.

Categories	Compound	C/EBPs	Diseases	Effect	References
Oligodeoxynucleotides	C/EBP*α*-saRNA	C/EBP*α*	Hepatocellular carcinoma	It upregulates C/EBP*α* protein in hepatocytes to inhibit cancer	[126]
	C/EBP*β*-dODN	C/EBP*β*	Hepatic fibrosis	C/EBP*β*-dODN inhibits the activation of C/EBP*β* and increases the activation of HSCs	[103]

Oligopeptides	Dominant negative C/EBP	C/EBP*α*, C/EBP*β*, C/EBP*δ*, C/EBP*ε*	Cardiac fibrosis	It inhibits the activations of C/EBP*α*, C/EBP*β*, C/EBP*δ*, and C/EBP*ε* and improves the cardiac fibrosis	[106]
	C/EBP*α*-DN	C/EBP*α*	Erythropoietic dysplasia	C/EBP*α*-DN suppresses the activation of C/EBP*α* in hematopoietic stem/progenitor cells	[384]
	A-C/EBP	C/EBP*β*	Related fat fibrosis	A-C/EBP inhibits the activation of C/EBP*β* in preadipocytes	[373]

Clinical medicines	Simvastatin	C/EBP*α*	Muscle fibrosis	Simvastatin inhibits the expression of C/EBP*α* protein and improves the muscle fibrosis	[346]
	Atorvastatin	C/EBP*β*	Cardiac fibrosis	Atorvastatin inhibits the expression of C/EBP*β* protein and improves the cardiac fibrosis	[294]
	Adefovir dipivoxil	C/EBP*β*	Hepatic fibrosis	Adefovir dipivoxil inhibits the expression of C/EBP*β* protein and improving the hepatic fibrosis	[148]
	Cortisol and dexamethasone	C/EBP*β*, C/EBP*δ*	Related lung fibrosis	Cortisol and dexamethasone enhance the activation of C/EBP*β* and C/EBP*δ* in the lung epithelial cells	[238]
	Mevastatin	C/EBP*δ*	Liver cancer	Mevastatin inhibits the expression of C/EBP*δ* protein in hepatoma cells	[164]
	Clenbuterol	C/EBP*δ*	Glioma	Clenbuterol increases the expression of C/EBP*δ* in glioma cells	[331]
	Atorvastatin	C/EBP*ζ*	Cardiac fibrosis	Atorvastatin inhibits the expression of C/EBP*ζ* and inhibiting the cardiac fibrosis	[385]
	Geranylgeranylacetone	C/EBP*ζ*	Hepatic fibrosis	Geranylgeranylacetone increases the expression of C/EBP*ζ* in HSCs and inhibiting the hepatic fibrosis	[386]
	Deferasirox	C/EBP*ζ*	Hepatic fibrosis	Deferasirox inhibits the expression of C/EBP*ζ* and inhibiting the hepatic fibrosis	[387]
	Candesartan	C/EBP*ζ*	Renal fibrosis	Candesartan inhibits the expression of C/EBP*ζ* and inhibiting the renal fibrosis	[281]
	Telmisartan	C/EBP*ζ*	Cardiac fibrosis	Telmisartan inhibits the expression of C/EBP*ζ* and inhibiting the cardiac fibrosis	[388]
	Metformin	C/EBP*ζ*	Intrauterine adhesion	Metformin inhibits the expression of C/EBP*ζ* and the intrauterine adhesion	[107]

Compounds	Baicalin	C/EBP*α*, C/EBP*β*, C/EBP*γ*, C/EBP*δ*, C/EBP*ζ*	Fat fibrosis	Baicalin inhibits the expression of C/EBP*α* protein and increases the expressions of C/EBP*β*, C/EBP*γ*, C/EBP*δ*, and C/EBP*ζ*	[389]
	5-Aza-dC	C/EBP*α*, C/EBP*β*, C/EBP*γ*	Hepatic fibrosis	5-Aza-dC inhibits the expressions of C/EBP*α*, C/EBP*β*, and C/EBP*γ*	[390]
	LPS	C/EBP*α*, C/EBP*δ*	Hepatic fibrosis	LPS increases the expressions of C/EBP*α* and C/EBP*δ* and inhibits the hepatic fibrosis	[28]
	Curcumin	C/EBP*α*	Hepatic fibrosis	Curcumin upregulates the expression of C/EBP*α* protein and inhibits the activation of HSCs	[374]
	Peretinoin	C/EBP*α*	Hepatic fibrosis	Peretinoin upregulates the expression of C/EBP*α* in hepatocytes and alleviates hepatic fibrosis	[128]
	Vitamin A	C/EBP*α*	Hepatic fibrosis	Vitamin A upregulates the expression of C/EBP*α* protein and inhibits the activation of HSCs	[117]
	Vitamin E	C/EBP*α*	Hepatic fibrosis	Vitamin E upregulates the expression of C/EBP*α* in hepatocytes and inhibits hepatic fibrosis	[136]
	Epigallocatechin-3-gallate	C/EBP*α*	Hepatic fibrosis	Vitamin E upregulates the expression of C/EBP*α* in hepatocytes and inhibits hepatic fibrosis	[115]
	Trichostatin A	C/EBP*α*	Hepatic fibrosis	Trichostatin A upregulates the expression of C/EBP*α* protein and inhibits the activation of HSCs	[122]
	BIX-01294	C/EBP*α*	Lung fibrosis	BIX-01294 upregulates the expression of C/EBP*α* protein and inhibits the activation of lung fibroblasts	[43]
	Echinomycin	C/EBP*β*	Fat fibrosis	Echinomycin inhibits the expression of C/EBP*β* protein and the adipogenesis	[356]
	Tanshinone IIA and Puerarin	C/EBP*β*	Cardiac fibrosis	Tanshinone IIA and Puerarin inhibit the expression of C/EBP*β* protein in macrophages and inhibit the cardiac fibrosis	[391]
	N-acetyl-Leu-Leu-norleucinal	C/EBP*β*	Lung fibrosis	N-acetyl-Leu-Leu-norleucinal blocks the activation of C/EBP*β* and inhibits the lung fibrosis	[392]
	Oltipraz	C/EBP*β*	Hepatic fibrosis	Oltipraz increases the activation of C/EBP*β* and inhibits the hepatic fibrosis	[103]
	Armepavine	C/EBP*β*	Hepatic fibrosis	Armepavine inhibits the activation of C/EBP*β* and inhibits the hepatic fibrosis	[393]
	8-O-cAMP	C/EBP*β*	Renal fibrosis	8-O-cAMP increases the expression and activation of C/EBP*β* and inhibits the renal fibrosis	[266]
	Chrysin	C/EBP*δ*	Neurological fibrosis	Chrysin inhibits the expression of C/EBP*δ* in microglial cells	[394]
	Ursolic acid	C/EBP*δ*	Renal fibrosis	Ursolic acid inhibits the expression of C/EBP*δ* protein and the kidney fibrosis	[348]
	Artesunate	C/EBP*ζ*	Intra-articular adhesion	Artesunate inhibits the expression of C/EBP*ζ* and the intra-articular adhesion	[395]
	Curcumin	C/EBP*ζ*	Lung fibrosis	Curcumin inhibits the expression of C/EBP*ζ* and the lung fibrosis	[250]
	Melatonin	C/EBP*ζ*	Hepatic fibrosis	Melatonin inhibits the expression of C/EBP*ζ* protein and the hepatic fibrosis	[396]
	Tauroursodeoxycholic acid	C/EBP*ζ*	Lung fibrosis	Tauroursodeoxycholic acid inhibits the expression of C/EBP*ζ* and the lung fibrosis	[169]
	4-Phenylbutyric acid	C/EBP*ζ*	Renal fibrosis	4-Phenylbutyric acid inhibits the expression of C/EBP*ζ* and the renal fibrosis	[283]
	Ginsenoside Rg1	C/EBP*ζ*	Renal fibrosis	Ginsenoside Rg1 inhibits the expression of C/EBP*ζ* and the renal fibrosis	[397]
	Quercetin	C/EBP*ζ*	Renal fibrosis	Quercetin inhibits the expression of C/EBP*ζ* and the renal fibrosis	[286]
	N-Acetyl-seryl-aspartyl-lysyl-proline	C/EBP*ζ*	Cardiac fibrosis	N-Acetyl-seryl-aspartyl-lysyl-proline inhibits the expression of C/EBP*ζ* and the cardiac fibrosis	[309]
	Apocynin	C/EBP*ζ*	Cardiac fibrosis	Apocynin inhibits the expression of C/EBP*ζ* and the cardiac fibrosis	[398]
	Execdin-4	C/EBP*ζ*	Cardiac fibrosis	Execdin-4 inhibits the expression of C/EBP*ζ* and the cardiac fibrosis	[399]
	Homoharringtonine	C/EBP*ζ*	Epidural Fibrosis	Homoharringtonine increases the expression of C/EBP*ζ* and inhibits the epidural fibrosis	[400]

## Abbreviations

ACE2: Angiotensin-converting enzyme 2
ADAM17: A disintegrin and metalloproteinase 17
ALS: Amyotrophic lateral sclerosis
AT1R: Angiotensin II type I receptor
Atg16L1: Autophagy-related 16 like 1
bZIP: Basic leucine zipper
CCl_4_: Carbon tetrachloride
CCL: C-C chemokine ligand
CCSP: Clara cell secretory protein
C/EBPs: CCAAT/enhancer-binding proteins
CFLAR: Fas-associated via death domain-like apoptosis regulator
CHOP: C/EBP homologous protein
CNS: Central nervous system
CTGF: Connective tissue growth factor
DBD: DNA-binding domain
DCM: Diabetic cardiomyopathy
DRP: Death receptor pathway
EAM: Experimental autoimmune myocarditis
ECM: Extracellular matrix
EGF: Epidermal growth factor
EMT: Epithelial-mesenchymal transition
ER: Endoplasmic reticulum
FANCD2: Fanconi anemia group D2 protein
FGF2: Fibroblast growth factor 2
FSP-1: Fibroblast-specific protein 1
GADD153: Growth arrest and DNA damage-inducible protein 153
HAS2: Hyaluronan synthase 2
HDAC1: Histone deacetylase 1
HIF-1*α*: Hypoxia-inducible factor-1*α*
HSC: Hepatic stellate cell
IFN: Interferon
IPF: Idiopathic pulmonary fibrosis
I/R: Ischemia-reperfusion
KLF5: Krüppel-like factor 5
LAP: Liver activation protein
LIP: Liver inhibitory protein
LPS: Lipopolysaccharide
LR-MSCs: Lung resident mesenchymal stem cells
MCD: Methionine-choline-deficient
MCP-1: Monocyte chemoattractant protein-1
MMP: Matrix metalloproteinase
MMT: Macrophage-to-myofibroblast transition
MP: Mitochondrial pathway
NASH: Nonalcoholic steatohepatitis
NGF: Nerve growth factor
P4HA1: Prolyl-4-hydroxylase alpha polypeptide 1
PAI-1: Plasminogen activator inhibitor-1
PDGF: Platelet-derived growth factor
PGC-1*α*: Peroxisome proliferator-activated receptor *γ* coactivator-1 alpha
PPAR*γ*: Peroxisome proliferator-activated receptor *γ*
PTMs: Posttranslational modifications
ROS: Reactive oxygen species
RSK: Ribosomal S6 kinase
saRNA: Short activating RNA
SCAR: Steatohepatitis-associated circRNA ATP5B regulator
SGD: Specific granule deficiency
SIAH2: Siah E3 ubiquitin protein ligase 2
SMAD: Mothers against decapentaplegic
SOCS3: Suppressor of cytokine signaling 3
SREBP1c: Sterol regulatory element-binding protein 1c
STAT3: Signal transducer and activator of transcription 3
SUMOylation: Small ubiquitin-like modifier modification
TAC: Transverse aortic constriction
TAD: Transcriptional activation domain
TECs: Tubular epithelial cells
TGF-*β*: Transforming growth factor-*β*
TNF-*α*: Tumor necrosis factor *α*
TREM1: Triggering receptor expressed on myeloid cells 1
TRIB1: Tribbles pseudokinase 1
uORF: Upstream open reading frame
UTR: Untranslated regions
UUO: Unilateral ureteral obstruction.

## Supplementary Materials

Supplementary Materials Table S1: the proteins interacted with C/EBPs and the functions.
