# Supplementary material for: CCAAT/Enhancer-Binding Proteins in Fibrosis: Complex Roles Beyond Conventional Understanding
Source: Research (Wash D C). 2022 Oct 3;2022:9891689. doi: 10.34133/2022/9891689 (PMC9575473; doi:10.34133/2022/9891689)
Supplement: Supplementary Materials — Table S1: the proteins interacted with C/EBPs and the functions. [file 9891689.f1.docx]

**Table S1. The proteins interacted with C/EBPs and the functions**

| **C/EBPs members** | **Types** | **Interacted partners** | **Functions** | **References** |
| --- | --- | --- | --- | --- |
| **C/EBPα** | **Transcription coregulatory factors** | C/EBP(α-ζ) | Interacts with different members of C/EBPs to regulate the expression of its target genes. | [1-3] |
|  |  | c-Jun | Interacts with C/EBPα to activate the endogenous PU.1 promoter | [4] |
|  |  | CREB/ATFs | Interacts with C/EBPα to regulate the expression of C/EBPα and ATFs target genes | [2] |
|  |  | DEK | Interacts with the dephosphorylated form of C/EBPα on S21 to regulate the transcription of gcsfr3 and cebpe | [5] |
|  |  | E2F | Interacts with C/EBPα to inhibit cell growth in myeloid cells | [6, 7] |
|  |  | FOXO1 | Interacts with C/EBPα to up-regulate adiponectin gene expression | [8, 9] |
|  |  | GR | Interacts with a p42, but not the p30 C/EBPα to upregulate p21((Waf1/Cip1)) expression | [10] |
|  |  | HDAC1 | Forms the HDAC1-C/EBPα complex to inhibit the transcription of telomerase reverse transcriptase(tert) gene | [11, 12] |
|  |  | HDAC3 | Interacts with C/EBPα to repress liver X receptor alpha (lxrα) transcription | [13] |
|  |  | KLF5 | Interacts and forms an complex to enhance the expression of s100a8 and s100a9 genes | [14] |
|  |  | NF-κB-p50 | Interacts with the C/EBPα bZIP domain for activation of the gene encoding bcl-2 | [15] |
|  |  | p300 | Binds with C/EBPα and acetylates it on K159, K250, K273, K275, and K276 to enhances its transactivation activity in Dgat1/2 gene; | [11, 16-18] |
|  |  | Schnurri2 | Interacts with Smad1/4 and C/EBPα to induce the expression of pparγ2 | [19] |
|  |  | SMAD3 | Interacts with C/EBPα and activates the mad1 promoter | [20] |
|  |  | SMAD1/4 | Interacts with C/EBPα to induce the expression of pparγ2 | [19, 21] |
|  |  | Tob2 | Interacts with C/EBPα to inhibit the C/EBPα recruitment to the pparγ2 promoter | [21] |
|  | **Enzymes** | CDK2 | Inhibits CDK2 activity by blocking the association of it with Cyclins to arrest cell proliferation | [7, 12, 22, 23] |
|  |  | CDK4 | Phosphorylates C/EBPα on S193 to decrease the binding of C/EBPα and C/EBPβ and enhance the complex formation of C/EBPα and HDAC1 or p300 | [7, 11, 16, 22] |
|  |  | ERK | Binds and phosphorylates C/EBPα on S21 to reduce its trans-activation activity | [24] |
|  |  | GSK3 | Phosphorylates C/EBPα on T222 and T226 to stimulate its its trans-activation activity | [25] |
|  |  | HTATIP | Interacts with C/EBPα to active the promoter of gcsfr | [26] |
|  |  | JNK | Interacts with C/EBPα to modulate the C/EBPα ubiquitination | [27] |
|  |  | MORC2 | Interacts with C/EBPα to promote C/EBPα sumoylation on K159 (K161 for human) and its subsequent degradation | [28] |
|  |  | p38 | Phosphorylates C/EBPα on S21 to inhibit the activity of C/EBPα and its ability to induce granulocyte differentiation | [29] |
|  |  | PKCδ | Phosphorylates C/EBPα on S247 to increase its trans-activation activity, and on S300 to attenuate its DNA-binding function | [30, 31] |
|  |  | PP2A | Mediates the dephosphorylation of C/EBPα on S193 | [7] |
|  |  | SIRT1 | Binds and de-acetylates C/EBPα to increase mitochondrial function | [18] |
|  |  | SUMO1 | Sumoylates C/EBPα on K159 to regulate the lung differentiation and the secretion of pulmonary surfactant proteins | [13, 32, 33] |
|  | **Others** | Beclin1 | Acetylation of lysine at positions K298, K302 and K326 of C/EBP-α promotes its binding to Beclin1 to induce the autophagy in activated hepatic stellate cells | [34] |
|  |  | Brm | Interacts with phosphorylated C/EBPα on S193 to sequestrate C/EBPα and then inhibit its trans-activation activity | [7, 12, 23, 35] |
|  |  | TGFβ2 | Sumoylation of C/EBPα is involved in the regulation of the interaction between C/EBPα and TGFβ2 in the lung | [36] |
|  |  | Trib2 | Binds the p42 C/EBPa and induces its ubiquitination on K314 (K313 for human) to induce its ubiquitin-dependent proteasome-mediated degradation | [37, 38] |
| **C/EBPβ** | **Transcription coregulatory factors** | C/EBP(α-ζ) | Interacts with different members of C/EBPs to regulate the expression of its target genes | [1-3] |
|  |  | CREB/ATFs | Interacts with C/EBPβ to regulate the expression of C/EBPβ and ATFs target genes | [2, 39] |
|  |  | DIPA | Binds C/EBPβ to inhibit its transcriptional activity | [40] |
|  |  | Fos/Jun | Interacts with the C/EBPα to induce the expression of endothelin A/B gene | [2, 41] |
|  |  | FKHR | Binds the C-terminal region of C/EBPβ to activate the promoter of dPRL | [42] |
|  |  | FOXO1A | Binds C/EBPβ to induce the transcription of decidual prolactin gene | [43] |
|  |  | FOXA3 | Interacts with C/EBPβ to enhance the transcription of pparγ2 gene | [44] |
|  |  | NF-κB-p50 | Interacts with the C/EBPα bZIP domain to activate NF-κB target genes | [15] |
|  |  | Runx2 | Binds C/EBPβ to activate the transcription of mmp-13 and hif-2α gene | [45] |
|  |  | SMAD3/4 | Interacts with C/EBPβ to repress the activity of C/EBPβ and then inhibit transcription from the PPAR2 and leptin promoters but not reduce the ability of C/EBPβ to bind to its cognate DNA sequence | [46] |
|  |  | SRF | Forms the C/EBPβ-SRF complex to interfere with SRF binding to the promoters of critical cardiac genes | [47, 48] |
|  |  | ZNF638 | Binds C/EBPβ to enhance the transcription of pparγ gene | [49] |
|  | **Enzymes** | CaMK | Binds C/EBPβ and phosphorylates it on S276 | [50] |
|  |  | CDK2 | Binds C/EBPβ and phosphorylates it on T235 for human | [51] |
|  |  | ERK | Binds C/EBPβ and phosphorylates it on T188 (T235 for human) | [48] |
|  |  | G9a | Binds and methylates C/EBPβ on K39 to interfere with activation of myeloid genes | [52] |
|  |  | GCN5 | Binds with C/EBPβ and acetylates it on K98/101/102 to dissociate it from C/EBPβ-HDAC1 complex and then enhance the transcription of C/EBPα promoter | [53] |
|  |  | GSK3β | Binds C/EBPβ and phosphorylates it on T179 and S184 to enhance its DNA binding activity and transcriptional activation | [54] |
|  |  | HADC1 | Binds and deacetylates C/EBPβ on K39 to inhibit the expression of cebpa, pparg, leptin, Glut4;  forms the C/EBPβ-HDAC1 complex to repress the transcription of dgat1/2 | [55-57] |
|  |  | p300 | Binds with and acetylates C/EBPβ on K39 to increase the expression of cebpa, pparg, leptin, Glut4; forms C/EBPβ/p300 complex to activate the DGAT1/2 promoters | [55, 56, 58] |
|  |  | p38 | Binds C/EBPβ and phosphorylates it on T188 to activate the binding of C/EBPβ to the atrogin1/MAFbx promoter | [59] |
|  |  | PKA/C | Binds C/EBPβ and phosphorylates it on S105, S223 (actually S222) and S240(actually S239) | [60] |
|  |  | PRMT4 | Binds and methylates C/EBPβ on R3 to interfere with recruitment of SWI/SNF to C/EBPβ | [61] |
|  |  | PRMT7 | Interacts with and methylates C/EBPβ to decrease the recruitment of it to the PPAR-γ2 promoter region and its nuclear fraction | [62] |
|  |  | RSK | Binds C/EBPβ and phosphorylates it on T217 to increase proliferation of stellate cells | [63] |
|  |  | SUMO1 | Binds and sumoylates C/EBPβ on K133 | [32] |
|  |  | SUMO2/3 | Binds and sumoylates C/EBPβ on K133 (K173, actually K174 for human) | [64] |
| **C/EBPγ** | **Transcription coregulatory factors** | ATF4 | Heterodimerizes with C/EBPγ to regulate the integrated stress response of cells | [65, 66] |
|  |  | ATF5 | Dimerizes with C/EBPγ to increase the transcription of vomeronasal 2 receptor gene | [67] |
|  |  | C/EBP(α-ζ) | Interacts with different members of C/EBPs to regulate the expression of its target genes | [1-3, 68] |
|  |  | YY1/E2F | Interact it to form C/EBPγ-YY1-E2F complex for increasing the transcription of ercc5 gene | [69] |
| **C/EBPδ** | **Transcription coregulatory factors** | C/EBP(α-ζ) | Interacts with different members of C/EBPs to regulate the expression of its target genes | [1-3] |
|  |  | CREB/ATFs | Interacts with C/EBPδ to regulate the expression of C/EBPδ and ATFs target genes | [2, 39] |
|  |  | DIPA | Binds C/EBPδ to inhibit its transcriptional activity | [40] |
|  |  | FOXA3 | Interacts with C/EBPδ to enhance the transcription of pparγ2 gene | [44] |
|  |  | GATA4 | Interacts with the bZIP domain of C/EBPδ to increase the expression of agp gene | [70] |
|  |  | Rad | Binds C/EBPδ to inhibit the binding of C/EBPδ to the CTGF promoter | [71] |
|  |  | Rb/E2F1 | C/EBPδ interacts with Rb and E2F1 to represses c-myc and cyclin e expression | [72] |
|  |  | Runx2 | Binds C/EBPδ to increase the transcriptional activity of osteocalcin II (OG2) promoter | [73, 74] |
|  |  | SMAD3/4 | Interacts with C/EBPδ to repress the activity of C/EBPδ and then inhibit transcription from the PPAR2 and leptin promoters but not reduce the ability of C/EBPδ to bind to its cognate DNA sequence | [46] |
|  |  | Sin3A | Binds the C-terminal of C/EBPδ | [75] |
|  |  | SP1 | Binds the bZIP domain of C/EBPδ to increase the transcription of il-10 gene | [76] |
|  |  | ZNF738 | Binds C/EBPδ to enhance the transcription of pparγ gene | [49] |
|  | **Enzymes** | CK2 | Binds and phosphorylates C/EBPδ on S57 to stimulate its transcriptional activity in the pparγ2 promoter | [77] |
|  |  | HDAC1 | Binds the N-terminal amino acid 36-164 domain of C/EBPδ to inhibit the transcriptional activity of haptoglobin promoter | [75] |
|  |  | GSK3β | Binds and phosphorylates C/EBPδ on T156 to enhance the degradation of it by FBXW7 | [78, 79] |
|  |  | p300 | Binds and acetylates C/EBPδ on K120 to enhance the cox-2 promoter activity | [80, 81] |
|  |  | PIAS1 | Binds the TAD of C/EBPδ to reduce its transcriptional activity but is independent of PIASy SUMO ligase activity | [81] |
|  |  | SENP1 | Binds and desumoylates C/EBPδ | [81] |
|  |  | SIAH2 | Binds and ubiquitinates C/EBPδ on K120 to promote its polyubiquitination and proteasomal degradation | [82] |
|  |  | SUMO1/2/3 | Binds and sumoylates C/EBPδ on K120 to abolish its interaction with p300 | [32, 80, 81] |
|  | **Others** | CBP | C/EBPδ interacts with CBP and trigger its phosphorylation | [83] |
|  |  | FANCD2 | C/EBPδ interacts with FANCD2 to chaperone it into the nucleus | [84] |
|  |  | FBXW7 | Binds C/EBPδ to enhance its polyubiquitination and proteasomal degradation | [79, 85] |
|  |  | IPO4 | Binds the NLS of C/EBPδ to augment its nuclear translocation | [84, 86] |
| **C/EBPε** | **Transcription coregulatory factors** | C/EBP(α-ζ) | Interacts with different members of C/EBPs to regulate the expression of its target genes | [1-3] |
|  |  | CREB/ATFs | Interacts with C/EBPε to regulate the expression of C/EBPε and ATFs target genes | [2, 87] |
|  |  | NF-κB-p65 | Binds C/EBPε to enhance its DNA binding on the lactoferrin and defensin promoter | [87] |
|  |  | Rb/E2F1 | C/EBPε binds them to repress the Rb-/E2F1-mediated transcriptional activity | [88] |
|  | **Enzymes** | AF17 | Interacts with C/EBPε | [89] |
|  |  | E6TP1 | Interacts with C/EBPε | [89] |
|  |  | ERK2 | Binds and phosphorylates C/EBPε | [18] |
|  |  | HDAC1 | Interacts with the acetylated C/EBPε on K121 and K198 to inhibit its transcriptional activity | [90] |
|  |  | LDOC1 | Interacts with C/EBPε | [89] |
|  |  | p300 | Binds and acetylates C/EBPε on K100, K121, K198 and K202 | [91] |
|  |  | p38 | Binds and phosphorylates C/EBPε on T75 for human(actually T74) to enhance the interaction between it and NF-κB-p65 | [18][87] |
|  |  | PIAS1 | Interacts with C/EBPε | [89] |
|  |  | PIASα/β | Interacts with C/EBPε to enhance its sumoylation on K121 | [92] |
|  |  | PKA | Binds and phosphorylates C/EBPε | [18] |
|  |  | Sirtuin 1 | Binds and deacetylates C/EBPε | [91] |
|  |  | SUMO1 | Binds and sumoylates C/EBPε on K121 | [32] |
|  |  | UBE2I | Interacts with C/EBPε | [89] |
|  |  | ZNF198 | Interacts with C/EBPε | [89] |
|  | **Others** | SMARCD2 | Interacts with C/EBPε result in recruitment it to the promoter of neutrophilic secondary granule genes and for granulocyte differentiation | [93, 94] |
|  |  | PML | Interacts with C/EBPε to activate its mediated transcription | [95] |
| **C/EBPζ**  **(CHOP)** | **Transcription coregulatory factors** | AP-1 | Binds the bZIP of C/EBPζ to activate promoter elements in the somatostatin, JunD, and collagenase genes | [96] |
|  |  | C/EBP(α-ε) | Interacts with other members of C/EBPs to regulate the expression of its target genes | [1-3] |
|  |  | CREB/ATFs | Interacts with C/EBPζ to regulate the expression of C/EBPζ and ATFs target genes | [2, 97] |
|  |  | FOXO3a | Interacts with C/EBPζ to induce the transcriptions of pro-apoptotic genes (puma and bim) | [98] |
|  |  | JUN | Interacts with C/EBPζ to form a complex that regulates the expression of TNFRSF10A/B | [99] |
|  | **Enzymes** | AMPKα1 | Binds and phosphorylates C/EBPζ on S30 in macrophages to trigger its degradation via ubiquitin-proteasome pathway | [100] |
|  |  | p38 | Binds and phosphorylates C/EBPζ on S78 and S81 (S79 and S82 for human) to enhance tis transactivation activity and is required for it induced apoptosis in macrophages | [101, 102] |
|  |  | SPOP | Interacts with C/EBPζ and triggers its degradation via the ubiquitin-proteasome pathway in prostate cancer | [103] |
|  |  | p300 | Binds the N-terminal domain of C/EBPζ to increase its ubiquitination and degradation | [104] |
|  |  | HDAC1/5/6 | Interacts with C/EBPζ to increase its degradation not involving the deacetylation of C/EBPζ itself | [104] |
|  |  | TRIM13 | Interacts with C/EBPζ to promote its ubiquitination and degradation | [105] |
|  | **Others** | Cyclophilin B | Interacts with the N-terminal domain of C/EBPζ and cooperates with p300 to modulate its ubiquitination | [106] |
|  |  | TRB3 | Interacts with the N-terminal domain of C/EBPζ to block the association of p300 and C/EBPζ | [104, 107] |

**References:**

1. J. Tsukada, Y. Yoshida, Y. Kominato, and P. E. Auron, “The CCAAT/enhancer (C/EBP) family of basic-leucine zipper (bZIP) transcription factors is a multifaceted highly-regulated system for gene regulation,” *Cytokine*, vol. 54, no. 1, pp. 6-19, 2011.
2. M. Pulido-Salgado, J. M. Vidal-Taboada, and J. Saura, “C/EBPbeta and C/EBPdelta transcription factors: Basic biology and roles in the CNS,” *Progress in Neurobiology*, vol. 132, pp. 1-33, 2015.
3. G. Grigoryan, A. W. Reinke, and A. E. Keating, “Design of protein-interaction specificity gives selective bZIP-binding peptides,” *Nature*, vol. 458, no. 7240, pp. 859-864, 2009.
4. D. H. Cai, D. Wang, J. Keefer, C. Yeamans, K. Hensley, and A. D. Friedman, “C/EBP alpha:AP-1 leucine zipper heterodimers bind novel DNA elements, activate the PU.1 promoter and direct monocyte lineage commitment more potently than C/EBP alpha homodimers or AP-1,” *Oncogene*, vol. 27, no. 19, pp. 2772-2779, 2008.
5. R. I. Koleva, S. B. Ficarro, H. S. Radomska et al., “C/EBPalpha and DEK coordinately regulate myeloid differentiation,” *Blood*, vol. 119, no.21, pp. 4878-4888, 2012.
6. B. A. Slomiany, K. L. D'Arigo, M. M. Kelly, and D. T. Kurtz, “C/EBPalpha inhibits cell growth via direct repression of E2F-DP-mediated transcription,” *BMC Molecular Biolog*y, vol. 20, no.16, pp. 5986-5997, 2000.
7. G. L. Wang, P. Iakova, M. Wilde, S. Awad, and N. A. Timchenko, “Liver tumors escape negative control of proliferation via PI3K/Akt-mediated block of C/EBP alpha growth inhibitory activity,” *Genes and Development*, vol. 18, no. 8, pp. 912-925, 2004.
8. L. Qiao, and J. Shao, “SIRT1 regulates adiponectin gene expression through Foxo1-C/enhancer-binding protein alpha transcriptional complex,” *Journal of Biological Chemistry*, vol. 281, no. 52, pp. 39915-39924, 2006.
9. K. Sekine, Y. R. Chen, N. Kojima, K. Ogata, A. Fukamizu, and A. Miyajima, “Foxo1 links insulin signaling to C/EBPalpha and regulates gluconeogenesis during liver development,” *EMBO Journal*, vol. 26, no. 15, pp. 3607-3615, 2007.
10. J. Q. Yang, J. J. Rudiger, J. M. Hughes et al., “Cell density and serum exposure modify the function of the glucocorticoid receptor C/EBP complex,” *American Journal of Respiratory Cell and Molecular Biology*, vol. 38, no. 4, pp. 414-422, 2008.
11. I. H. Hong, K. Lewis, P. Iakova et al., “Age-associated change of C/EBP family proteins causes severe liver injury and acceleration of liver proliferation after CCl4 treatments,” *Journal of Biological Chemistry*, vol. 289, no. 2, pp. 1106-1118, 2014.
12. J. Jin, G. L. Wang, P. Iakova et al., “Epigenetic changes play critical role in age-associated dysfunctions of the liver,” *Aging Cell*, vol. 9, no. 5, pp. 895-910, 2010.
13. J. Ren, D. Li, Y. Li et al., “HDAC3 interacts with sumoylated C/EBPalpha to negatively regulate the LXRalpha expression in rat hepatocytes,” *Molecular and Cellular Endocrinology*, vol. 374, no. 1-2, pp. 35-45, 2013.
14. K. Fujiu, I. Manabe, and R. Nagai, “Renal collecting duct epithelial cells regulate inflammation in tubulointerstitial damage in mice,” *Journal of Clinical Investigation*, vol. 121, no. 9, pp. 3425-3441, 2011.
15. J. E. Dooher, I. Paz-Priel, S. Houng, A. S. Baldwin Jr, and A. D. Friedman, “C/EBPalpha, C/EBPalpha oncoproteins, or C/EBPbeta preferentially bind NF-kappaB p50 compared with p65, focusing therapeutic targeting on the C/EBP:p50 interaction,” *Molecular Cancer Research*, vol. 9, no. 10, pp. 1395-1405, 2011.
16. J. Jin, L. Valanejad, T. P. Nguyen et al., “Activation of CDK4 Triggers Development of Non-alcoholic Fatty Liver Disease,” *Cell Reports*, vol. 16, no. 3, pp. 744-756, 2016.
17. B. Guillory, N. Jawanmardi, P. Iakova et al., “Ghrelin deletion protects against age-associated hepatic steatosis by downregulating the C/EBPalpha-p300/DGAT1 pathway,” *Aging Cell*, vol. 17, no. 1, pp. e12688, 2018.
18. M. A. Zaini, C. Muller, T. V. de Jong et al., “A p300 and SIRT1 Regulated Acetylation Switch of C/EBPalpha Controls Mitochondrial Function,” *Cell Reports*, vol. 22, no. 2, pp. 497-511, 2018.
19. W. Jin, T. Takagi, S. N. Kanesashi et al., “Schnurri-2 controls BMP-dependent adipogenesis via interaction with Smad proteins,” *Developmental Cell*, vol. 10, no. 4, pp. 461-471, 2006.
20. N. Hein, K. Jiang, C. Cornelissen, and B. Luscher, “TGFbeta1 enhances MAD1 expression and stimulates promoter-bound Pol II phosphorylation: basic functions of C/EBP, SP and SMAD3 transcription factors,” *BMC Molecular Biology*, vol. 12, pp. 9, 2011.
21. A. Takahashi, M. Morita, K. Yokoyama, T. Suzuki, and T. Yamamoto, “Tob2 inhibits peroxisome proliferator-activated receptor gamma2 expression by sequestering Smads and C/EBPalpha during adipocyte differentiation,” *Molecular and Cellular Biology*, vol. 32, no 24., pp. 5067-5077, 2012.
22. H. Wang, P. Iakova, M. Wilde et al., “C/EBPalpha arrests cell proliferation through direct inhibition of Cdk2 and Cdk4,” *Molecular Cell*, vol. 8, no. 4, pp. 817-828, 2001.
23. G. L. Wang, X. Shi, E. Salisbury et al., “Cyclin D3 maintains growth-inhibitory activity of C/EBPalpha by stabilizing C/EBPalpha-cdk2 and C/EBPalpha-Brm complexes,” *Molecular and Cellular Biology*, vol. 26, no. 7, pp. 2570-2582, 2006.
24. G. D. Jack, L. Zhang, and A. D. Friedman, “M-CSF elevates c-Fos and phospho-C/EBPalpha(S21) via ERK whereas G-CSF stimulates SHP2 phosphorylation in marrow progenitors to contribute to myeloid lineage specification,” *Blood*, vol. 114, no. 10, pp. 2172-2180, 2009.
25. S. E. Ross, R. L. Erickson, N. Hemati, and O. A. MacDougald, “Glycogen synthase kinase 3 is an insulin-regulated C/EBPalpha kinase,” *Molecular and Cellular Biology*, vol. 19, no. 12, pp. 8433-8441, 1999.
26. D. Bararia, A. K. Trivedi, A. A. Zada et al., “Proteomic identification of the MYST domain histone acetyltransferase TIP60 (HTATIP) as a co-activator of the myeloid transcription factor C/EBPalpha,” *Leukemia*, vol. 22, no. 4, pp. 800-807, 2008.
27. A. K. Trivedi, D. Bararia, M. Christopeit et al., “Proteomic identification of C/EBP-DBD multiprotein complex: JNK1 activates stem cell regulator C/EBPalpha by inhibiting its ubiquitination,” *Oncogene*, vol. 26, no. 12, pp. 1789-1801, 2007.
28. J. Liu, Q. Zhang, B. Ruan et al., “MORC2 regulates C/EBPalpha-mediated cell differentiation via sumoylation,” *Cell Death and Differentiation*, vol. 26, no. 10, pp. 1905-1917, 2019.
29. C. R. Geest, M. Buitenhuis, A. G. Laarhoven et al., “p38 MAP kinase inhibits neutrophil development through phosphorylation of C/EBPalpha on serine 21,” *Stem Cells*, vol. 27, no. 9, pp. 2271-2282, 2009.
30. G. Behre, S. M. Singh, H. Liu et al., “Ras signaling enhances the activity of C/EBP alpha to induce granulocytic differentiation by phosphorylation of serine 248,” *Journal of Biological Chemistry*, vol. 277, no. 29, pp. 26293-26299, 2002.
31. C. W. Mahoney, J. Shuman, S. L. McKnight, H. C. Chen, and K. P. Huang, “Phosphorylation of CCAAT-enhancer binding protein by protein kinase C attenuates site-selective DNA binding,” *Journal of Biological Chemistry*, vol. 267, no. 27, pp. 19396-19403, 1992.
32. J. Kim, C. A. Cantwell, P. F. Johnson, C. M. Pfarr, and S. C. Williams, “Transcriptional activity of CCAAT/enhancer-binding proteins is controlled by a conserved inhibitory domain that is a target for sumoylation,” *Journal of Biological Chemistry*, vol. 277, no. 41, pp. 38037-38044, 2002.
33. Y. D. Chen, J. Y. Liu, Y. M. Lu et al., “Functional roles of C/EBPalpha and SUMOmodification in lung development,” *International Journal of Molecular Medicine*, vol. 40, no. 4, pp. 1037-1046, 2017.
34. C. Hou, S. Lu, Y. Su et al., “C/EBP-alpha induces autophagy by binding to Beclin1 through its own acetylation modification in activated hepatic stellate cells,” *Experimental Cell Research*, vol. 405, no. 2, pp. 112721, 2021.
35. G. L. Wang, X. Shi, E. Salisbury et al., “Growth hormone corrects proliferation and transcription of phosphoenolpyruvate carboxykinase in livers of old mice via elimination of CCAAT/enhancer-binding protein alpha-Brm complex,” *Journal of Biological Chemistry*, vol. 282, no. 2, pp. 1468-1478, 2007.
36. Y. Zhu, X. Chen, L. Mi et al., “Sumoylation of CCAAT-enhancer-binding protein alpha inhibits lung differentiation in Bronchopulmonary Dysplasia model rats,” *Journal of Cellular and Molecular Medicine*, vol. 24, no 12., pp. 7067-7071, 2020.
37. K. Keeshan, Y. He, B. J. Wouters et al., “Tribbles homolog 2 inactivates C/EBPalpha and causes acute myelogenous leukemia,” *Cancer Cell*, vol. 10, no. 5, pp. 401-411, 2006.
38. C. O'Connor, F. Lohan, J. Campos et al., “The presence of C/EBPalpha and its degradation are both required for TRIB2-mediated leukaemia,” *Oncogene*, vol. 35, no. 40, pp. 5272-5281, 2016.
39. X. Sun, P. Jefferson, Q. Zhou, J. M. Angelastro, and L. A. Greene, “Dominant-Negative ATF5 Compromises Cancer Cell Survival by Targeting CEBPB and CEBPD,” *Molecular Cancer Research*, vol. 18, no. 2, pp. 216-228, 2020.
40. O. Bezy, C. Elabd, O. Cochet et al., “Delta-interacting protein A, a new inhibitory partner of CCAAT/enhancer-binding protein beta, implicated in adipocyte differentiation,” *Journal of Biological Chemistry*, vol. 280, no. 12, pp. 11432-11438, 2005.
41. J. Wang, H. Y. Ma, R. R. Krishnamoorthy, T. Yorio, and S. He, “A feed-forward regulation of endothelin receptors by c-Jun in human non-pigmented ciliary epithelial cells and retinal ganglion cells,” *Public Library of Science One*, vol. 12, no. 9, pp. e0185390, 2017.
42. M. Christian, X. Zhang, T. Schneider-Merck et al., “Cyclic AMP-induced forkhead transcription factor, FKHR, cooperates with CCAAT/enhancer-binding protein beta in differentiating human endometrial stromal cells,” *Journal of Biological Chemistry*, vol. 277, no. 23, pp. 20825-20832, 2002.
43. V. J. Lynch, G. May, and G. P. Wagner, “Regulatory evolution through divergence of a phosphoswitch in the transcription factor CEBPB,” *Nature*, vol. 480, no. 7377, pp. 383-386, 2011.
44. L. Xu, V. Panel, X. Ma et al., “The winged helix transcription factor Foxa3 regulates adipocyte differentiation and depot-selective fat tissue expansion,” *Molecular and Cellular Biology*, vol. 33, no. 17, pp. 3392-3399, 2013.
45. M. Hirata, F. Kugimiya, A. Fukai et al., “C/EBPbeta and RUNX2 cooperate to degrade cartilage with MMP-13 as the target and HIF-2alpha as the inducer in chondrocytes,” *Human Molecular Genetics*, vol. 21, no. 5, pp. 1111-1123, 2012.
46. L. Choy, and R. Derynck, “Transforming growth factor-beta inhibits adipocyte differentiation by Smad3 interacting with CCAAT/enhancer-binding protein (C/EBP) and repressing C/EBP transactivation function,” *Journal of Biological Chemistry*, vol. 278, no. 11, pp. 9609-9619, 2003.
47. P. Bostrom, N. Mann, J. Wu et al., “C/EBPbeta controls exercise-induced cardiac growth and protects against pathological cardiac remodeling,” *Cell*, vol. 143, no. 7, pp. 1072-1083, 2010.
48. M. Hanlon, T. W. Sturgill, and L. Sealy, “ERK2- and p90(Rsk2)-dependent pathways regulate the CCAAT/enhancer-binding protein-beta interaction with serum response factor,” *Journal of Biological Chemistry*, vol. 276, no. 42, pp. 38449-38456, 2001.
49. S. Meruvu, L. Hugendubler, and E. Mueller, “Regulation of adipocyte differentiation by the zinc finger protein ZNF638,” *Journal of Biological Chemistry*, vol. 286, no. 30, pp. 26516-26523, 2011.
50. M. Wegner, Z. Cao, and M. G. Rosenfeld, “Calcium-regulated phosphorylation within the leucine zipper of C/EBP beta,” *Science*, vol. 256, no. 5055, pp. 370-373, 1992.
51. X. Li, J. W. Kim, M. Gronborg, H. Urlaub, M. D. Lane, and Q. Q. Tang, “Role of cdk2 in the sequential phosphorylation/activation of C/EBPbeta during adipocyte differentiation,” *Proceedings of the National Academy of Sciences of the United States of America*, vol. 104, no. 28, pp. 11597-11602, 2007.
52. A. Leutz, O. Pless, M. Lappe, G. Dittmar, and E. Kowenz-Leutz, “Crosstalk between phosphorylation and multi-site arginine/lysine methylation in C/EBPs,” *Transcription*, vol. 2, no. 1, pp. 3-8, 2011.
53. N. Wiper-Bergeron, H. A. Salem, J. J. Tomlinson, D. Wu, and R. J. Hache, “Glucocorticoid-stimulated preadipocyte differentiation is mediated through acetylation of C/EBPbeta by GCN5,” *Proceedings of the National Academy of Sciences of the United States of America*, vol. 104, no. 8, pp. 2703-2708, 2007.
54. J. W. Kim, Q. Q. Tang, X. Li, and M. D. Lane, “Effect of phosphorylation and S-S bond-induced dimerization on DNA binding and transcriptional activation by C/EBPbeta,” *Proceedings of the National Academy of Sciences of the United States of America*, vol. 104, no. 6, pp. 1800-1804, 2007.
55. K. Lewis, L. Valanejad, A. Cast et al., “RNA Binding Protein CUGBP1 Inhibits Liver Cancer in a Phosphorylation-Dependent Manner,” *Molecular and Cellular Biology*, vol. 37, no.16, pp. e00128-17, 2017.
56. T. I. Cesena, T. X. Cui, L. Subramanian et al., “Acetylation and deacetylation regulate CCAAT/enhancer binding protein beta at K39 in mediating gene transcription,” *Molecular and Cellular Endocrinology*, vol. 289, no. 1-2, pp. 94-101, 2008.
57. Y. Jiang, P. Iakova, J. Jin et al., “Farnesoid X receptor inhibits gankyrin in mouse livers and prevents development of liver cancer,” *Hepatology*, vol. 57, no. 3, pp. 1098-1106, 2013.
58. Z. Liu, C. Li, N. Kang, H. Malhi, V. H. Shah, and J. L. Maiers, “Transforming growth factor beta (TGFbeta) cross-talk with the unfolded protein response is critical for hepatic stellate cell activation,” *Journal of Biological Chemistry*, vol. 294, no. 9, pp. 3137-3151, 2019.
59. G. Zhang, B. Jin, and Y. P. Li, “C/EBPbeta mediates tumour-induced ubiquitin ligase atrogin1/MAFbx upregulation and muscle wasting,” *EMBO Journal*, vol. 30, no. 20, pp. 4323-4335, 2011.
60. C. Trautwein, P. van der Geer, M. Karin, T. Hunter, and M. Chojkier, “Protein kinase A and C site-specific phosphorylations of LAP (NF-IL6) modulate its binding affinity to DNA recognition elements,” *Journal of Clinical Investigation*, vol. 93, no. 6, pp. 2554-2561, 1994.
61. E. Kowenz-Leutz, O. Pless, G. Dittmar, M. Knoblich, and A. Leutz, “Crosstalk between C/EBPbeta phosphorylation, arginine methylation, and SWI/SNF/Mediator implies an indexing transcription factor code,” *EMBO Journal*, vol. 29, no. 6, pp. 1105-1115, 2010.
62. Y. E. Leem, J. H. Bae, H. J. Jeong, and J. S. Kang, “PRMT7 deficiency enhances adipogenesis through modulation of C/EBP-beta,” *Biochemical and Biophysical Research Communications*, vol. 517, no. 3, pp. 484-490, 2019.
63. M. Buck, V. Poli, T. Hunter, and M. Chojkier, “C/EBPbeta phosphorylation by RSK creates a functional XEXD caspase inhibitory box critical for cell survival,” *Molecular Cell*, vol. 8, no. 4, pp. 807-816, 2001.
64. E. M. Eaton, and L. Sealy, “Modification of CCAAT/enhancer-binding protein-beta by the small ubiquitin-like modifier (SUMO) family members, SUMO-2 and SUMO-3,” *Journal of Biological Chemistry*, vol. 278, no. 35, pp. 33416-33421, 2003.
65. I. K. Mann, R. Chatterjee, J. Zhao et al., “CG methylated microarrays identify a novel methylated sequence bound by the CEBPB|ATF4 heterodimer that is active in vivo,” *Genome Research*, vol. 23, no. 6, pp. 988-997, 2013.
66. C. J. Huggins, M. K. Mayekar, N. Martin et al., “C/EBPgamma Is a Critical Regulator of Cellular Stress Response Networks through Heterodimerization with ATF4,” *Molecular and Cellular Biology*, vol. 36, no. 5, pp. 693-713, 2015.
67. H. Nakano, Y. Iida, T. Murase et al., “Co-expression of C/EBPgamma and ATF5 in mouse vomeronasal sensory neurons during early postnatal development,” *Cell and Tissue Research*, vol. 378, no. 3, pp. 427-440, 2019.
68. C. J. Huggins, R. Malik, S. Lee et al., “C/EBPgamma suppresses senescence and inflammatory gene expression by heterodimerizing with C/EBPbeta,” *Molecular and Cellular Biology*, vol. 33, no. 16, pp. 3242-3258, 2013.
69. E. L. Crawford, T. Blomquist, D. N. Mullins et al., “CEBPG regulates ERCC5/XPG expression in human bronchial epithelial cells and this regulation is modified by E2F1/YY1 interactions,” *Carcinogenesis*, vol. 28, no. 12, pp. 2552-2559, 2007.
70. N. Turgeon, D. Rousseau, E. Roy, and C. Asselin, “GATA-4 modulates C/EBP-dependent transcriptional activation of acute phase protein genes,” *Biochemical and Biophysical Research Communications*, vol. 370, no. 2, pp. 371-375, 2008.
71. J. Zhang, L. Chang, C. Chen et al., “Rad GTPase inhibits cardiac fibrosis through connective tissue growth factor,” *Cardiovascular Research*, vol. 91, no. 1, pp. 90-98, 2011.
72. S. Gery, S. Tanosaki, W. K. Hofmann, A. Koppel, and H. P. Koeffler, “C/EBPdelta expression in a BCR-ABL-positive cell line induces growth arrest and myeloid differentiation,” *Oncogene*, vol. 24, no. 9, pp. 1589-1597, 2005.
73. T. L. McCarthy, C. Ji, Y. Chen et al., “Runt domain factor (Runx)-dependent effects on CCAAT/ enhancer-binding protein delta expression and activity in osteoblasts,” *Journal of Biological Chemistry*, vol. 275, no. 28, pp. 21746-21753, 2000.
74. C. S. Shin, M. J. Jeon, J. Y. Yang et al., “CCAAT/enhancer-binding protein delta activates the Runx2-mediated transcription of mouse osteocalcin II promoter,” *Journal of Molecular Endocrinology*, vol. 36, no. 3, pp. 531-546, 2006.
75. N. Turgeon, C. Valiquette, M. Blais, S. Routhier, E. G. Seidman, and C. Asselin, “Regulation of C/EBPdelta-dependent transactivation by histone deacetylases in intestinal epithelial cells,” *Journal of Cellular Biochemistry*, vol. 103, no. 5, pp. 1573-1583, 2008.
76. B. T. Chiang, Y. W. Liu, B. K. Chen, J. M. Wang, and W. C. Chang, “Direct interaction of C/EBPdelta and Sp1 at the GC-enriched promoter region synergizes the IL-10 gene transcription in mouse macrophage,” *Journal of Biomedical Science*, vol. 13, no. 5, pp. 621-635, 2006.
77. L. Schwind, A. D. Zimmer, C. Gotz, and M. Montenarh, “CK2 phosphorylation of C/EBPdelta regulates its transcription factor activity,” *International Journal of Biochemistry and Cell Biology*, vol. 61, pp. 81-89, 2015.
78. K. Balamurugan, and E. Sterneck, “The many faces of C/EBPdelta and their relevance for inflammation and cancer,” *International journal of biological sciences*, vol. 9, no. 9, pp. 917-933, 2013
79. K. Balamurugan, S. Sharan, K. D. Klarmann et al., “FBXW7alpha attenuates inflammatory signalling by downregulating C/EBPdelta and its target gene Tlr4,” *Nature Communications*, vol. 4, pp. 1662, 2013.
80. J. M. Wang, C. Y. Ko, L. C. Chen, W. L. Wang, and W. C. Chang, “Functional role of NF-IL6beta and its sumoylation and acetylation modifications in promoter activation of cyclooxygenase 2 gene,” *Nucleic Acids Research*, vol. 34, no. 1, pp. 217-231, 2006.
81. S. Zhou, J. Si, T. Liu, and J. W. DeWille, “PIASy represses CCAAT/enhancer-binding protein delta (C/EBPdelta) transcriptional activity by sequestering C/EBPdelta to the nuclear periphery,” *Journal of Biological Chemistry*, vol. 283, no. 29, pp. 20137-20148, 2008.
82. T. R. Sarkar, S. Sharan, J. Wang et al., “Identification of a Src tyrosine kinase/SIAH2 E3 ubiquitin ligase pathway that regulates C/EBPdelta expression and contributes to transformation of breast tumor cells,” *Molecular and Cellular Biology*, vol. 32, no. 2, pp. 320-332, 2012.
83. K. A. Kovacs, M. Steinmann, P. J. Magistretti, O. Halfon, and J. R. Cardinaux, “CCAAT/enhancer-binding protein family members recruit the coactivator CREB-binding protein and trigger its phosphorylation,” *Journal of Biological Chemistry*, vol. 278, no. 38, pp. 36959-36965, 2003.
84. J. Wang, T. R. Sarkar, M. Zhou et al., “CCAAT/enhancer binding protein delta (C/EBPdelta, CEBPD)-mediated nuclear import of FANCD2 by IPO4 augments cellular response to DNA damage,” *Proceedings of the National Academy of Sciences of the United States of America*, vol. 107, no. 37, pp. 16131-16136, 2010.
85. D. Mendoza-Villanueva, K. Balamurugan, H. R. Ali et al., “The C/EBPdelta protein is stabilized by estrogen receptor alpha activity, inhibits SNAI2 expression and associates with good prognosis in breast cancer,” *Oncogene*, vol. 35, no. 48, pp. 6166-6176, 2016.
86. Y. Zhou, F. Liu, Q. Xu et al., “Inhibiting Importin 4-mediated nuclear import of CEBPD enhances chemosensitivity by repression of PRKDC-driven DNA damage repair in cervical cancer,” *Oncogene*, vol. 39, no. 34, pp. 5633-5648, 2020.
87. A. M. Chumakov, A. Silla, E. A. Williamson, and H. P. Koeffler, “Modulation of DNA binding properties of CCAAT/enhancer binding protein epsilon by heterodimer formation and interactions with NFkappaB pathway,” *Blood*, vol. 109, no. 10, pp. 4209-4219, 2007.
88. S. Gery, A. F. Gombart, Y. K. Fung, and H. P. Koeffler, “C/EBPepsilon interacts with retinoblastoma and E2F1 during granulopoiesis,” *Blood*, vol. 103, no. 3, pp. 828-835, 2004.
89. D. Y. Chih, D. J. Park, M. Gross et al., “Protein partners of C/EBPepsilon,” *Experimental Hematology*, vol. 32, no. 12, pp. 1173-1181, 2004.
90. M. Muraoka, T. Akagi, A. Ueda et al., “C/EBPepsilon DeltaRS derived from a neutrophil-specific granule deficiency patient interacts with HDAC1 and its dysfunction is restored by trichostatin A,” *Biochemical and Biophysical Research Communications*, vol. 516, no. 1, pp. 293-299, 2019.
91. M. Bartels, A. M. Govers, V. Fleskens et al., “Acetylation of C/EBPepsilon is a prerequisite for terminal neutrophil differentiation,” *Blood*, vol. 125, no. 11, pp. 1782-1792, 2015.
92. J. Kim, S. Sharma, Y. Li, E. Cobos, J. J. Palvimo, and S. C. Williams, “Repression and coactivation of CCAAT/enhancer-binding protein epsilon by sumoylation and protein inhibitor of activated STATx proteins,” *Journal of Biological Chemistry*, vol. 280, no. 13, pp. 12246-12254, 2005.
93. P. Priam, V. Krasteva, P. Rousseau et al., “SMARCD2 subunit of SWI/SNF chromatin-remodeling complexes mediates granulopoiesis through a CEBPvarepsilon dependent mechanism,” *Nature Genetics*, vol. 49, no. 5, pp. 753-764, 2017.
94. M. Witzel, D. Petersheim, Y. Fan et al., “Chromatin-remodeling factor SMARCD2 regulates transcriptional networks controlling differentiation of neutrophil granulocytes,” *Nature Genetics*, vol. 49, no. 5, pp. 742-752, 2017.
95. Y. Tagata, H. Yoshida, L. A. Nguyen et al., “Phosphorylation of PML is essential for activation of C/EBP epsilon and PU.1 to accelerate granulocytic differentiation,” *Leukemia*, vol. 22, no. 2, pp. 273-280, 2008.
96. M. Ubeda, M. Vallejo, and J. F. Habener, “CHOP enhancement of gene transcription by interactions with Jun/Fos AP-1 complex proteins,” *Molecular and Cellular Biology*, vol. 19, no. 11, pp. 7589-7599, 1999.
97. N. Ohoka, S. Yoshii, T. Hattori, K. Onozaki, and H. Hayashi, “TRB3, a novel ER stress-inducible gene, is induced via ATF4-CHOP pathway and is involved in cell death,” *EMBO Journal*, vol. 24, no. 6, pp. 1243-1255, 2005.
98. A. P. Ghosh, B. J. Klocke, M. E. Ballestas, and K. A. Roth, “CHOP potentially co-operates with FOXO3a in neuronal cells to regulate PUMA and BIM expression in response to ER stress,” *Public Library of Science One*, vol. 7, no. 6, pp. e39586, 2012.
99. T. Li, L. Su, Y. Lei, X. Liu, Y. Zhang, and X. Liu, “DDIT3 and KAT2A Proteins Regulate TNFRSF10A and TNFRSF10B Expression in Endoplasmic Reticulum Stress-mediated Apoptosis in Human Lung Cancer Cells,” *Journal of Biological Chemistry*, vol. 290, no. 17, pp. 11108-11118, 2015.
100. X. Dai, Y. Ding, Z. Liu, W. Zhang, and M. H. Zou, “Phosphorylation of CHOP (C/EBP Homologous Protein) by the AMP-Activated Protein Kinase Alpha 1 in Macrophages Promotes CHOP Degradation and Reduces Injury-Induced Neointimal Disruption In Vivo,” *Circulation Research*, vol. 119, no. 10, pp. 1089-1100, 2016.
101. X. Z. Wang, and D. Ron, “Stress-induced phosphorylation and activation of the transcription factor CHOP (GADD153) by p38 MAP Kinase,” *Science*, vol. 272, no. 5266, pp. 1347-1349, 1996.
102. T. Devries-Seimon, Y. Li, P. M. Yao et al., “Cholesterol-induced macrophage apoptosis requires ER stress pathways and engagement of the type A scavenger receptor,” *Journal of Cell Biology*, vol. 171, no. 1, pp. 61-73, 2005.
103. P. Zhang, K. Gao, Y. Tang et al., “Destruction of DDIT3/CHOP protein by wild-type SPOP but not prostate cancer-associated mutants,” *Human Mutation*, vol. 35, no. 9, pp. 1142-1151, 2014.
104. N. Ohoka, T. Hattori, M. Kitagawa, K. Onozaki, and H. Hayashi, “Critical and functional regulation of CHOP (C/EBP homologous protein) through the N-terminal portion,” *Journal of Biological Chemistry*, vol. 282, no. 49, pp. 35687-35694, 2007.
105. Y. Li, D. Ren, Y. Shen, X. Zheng, and G. Xu, “Altered DNA methylation of TRIM13 in diabetic nephropathy suppresses mesangial collagen synthesis by promoting ubiquitination of CHOP,” *EBioMedicine,* vol. 51, pp. 102582, 2020.
106. K. Jeong, H. Kim, K. Kim et al., “Cyclophilin B is involved in p300-mediated degradation of CHOP in tumor cell adaptation to hypoxia,” *Cell Death and Differentiation*, vol. 21, no. 3, pp. 438-450, 2014.
107. Y. Yang, L. Liu, I. Naik, Z. Braunstein, J. Zhong, and B. Ren, “Transcription Factor C/EBP Homologous Protein in Health and Diseases,” *Frontiers in Immunology*, vol. 8, pp. 1612, 2017.
